# SFRP2 mediates Epstein-Barr virus and bladder cancer risk: a Mendelian randomization study and colocalization analysis

**DOI:** 10.1038/s41598-025-91594-9

**Published:** 2025-02-28

**Authors:** Jian Li, Bing Yang, Lei Guo, Wenqi Huang, Qiong Hu, Hongting Yan, Changpu Du, Rong Tan, Dongxin Tang

**Affiliations:** 1 The First College of Clinical Medicine, Guizhou University of Traditional Chinese Medicine, Guiyang, Guizhou China; 2 Student Management Office, The First Affiliated Hospital of Guizhou University of Traditional Chinese Medicine, Guiyang, Guizhou China; 3 Department of Geratology, The First Affiliated Hospital of Guizhou University of Traditional Chinese Medicine, Guiyang, Guizhou Province China; 4 Department of Oncology, The First Affiliated Hospital of Guizhou University of Traditional Chinese Medicine, Guiyang, Guizhou Province China; 5 Department of Pharmaceutics, The First Affiliated Hospital of Guizhou University of Traditional Chinese Medicine, No. 71 Bao Shan North Road, Yunyan District, Guiyang, 550001 Guizhou China; 6 Guizhou University of Traditional Chinese Medicine, No. 4, Dongqing Road, Huaxi District, Guiyang, 550025 Guizhou China

**Keywords:** Epstein-Barr virus, Bladder cancer, Secreted frizzled-related protein, Causal association, Mendelian randomization, Cancer epidemiology, Cancer genetics, Bladder cancer, Risk factors

## Abstract

Studies suggest a possible association between Epstein-Barr virus (EBV) infection and bladder cancer (BCa) risk, though this remains unclear. Secreted frizzled-related protein (sFRP) is also linked to BCa, with some DNA viruses potentially regulating its expression. This study used Mendelian randomization (MR) and colocalization analysis to explore the causal relationship between EBV infection, BCa risk, and the mediating role of sFRP. We first performed a two-sample MR study to assess the causal relationship between 5 EBV-related antibodies (AEB-IgG, EA-D, EBNA-1, VCA-p18, ZEBRA) and BCa using the Finnish Consortium’s R11 dataset, validated with R10. Reverse MR analysis followed. For significant results, multivariable MR (MVMR) was applied to adjust for confounding risk factors. A two-step MR explored the potential mediating role of 3 sFRPs (sFRP1, sFRP2, sFRP3) between positive exposures and BCa. Colocalization analysis were conducted for positive exposures, mediators, and BCa, with multiple sensitivity analyses confirming the robustness of the results. The two-sample Mendelian randomization study found that EBNA-1 (OR = 1.15, 95% CI: 1.01–1.30; *p* = 0.039) and VCA-p18 (OR = 1.36, 95% CI: 1.13–1.64; *p* = 0.001) may increase BCa risk, with only VCA-p18 (P_fdr = 0.006) showing a significant effect after False Discovery Rate (FDR) correction. The Finnish Consortium R10 replication study yielded similar results, and reverse MR analysis did not suggest reverse causality. After MVMR adjusted for relevant confounders, VCA-p18 (OR = 1.40, 95% CI: 1.13–1.74; *p* = 0.002) still significantly increased BCa risk. Two-step MR identified sFRP2 as a mediator, with VCA-p18 down-regulating sFRP2 expression to elevate BCa risk. Colocalization analysis suggested a shared causal variant (nearby gene HLA-DQA1) between VCA-p18 and BCa (PPH4 = 65.44%). Multiple sensitivity analyses confirmed the robustness of the results. Our study suggests that EBV infection (VCA-p18 antibody) may increase the risk of BCa by lowering sFRP2 levels. Additionally, EBNA-1 antibodies may also contribute to an elevated risk of BCa. We hope these findings will provide new insights for future research on the association between EBV and BCa.

**Supplementary Information:**

The online version contains supplementary material available at 10.1038/s41598-025-91594-9.

## Introduction

Bladder cancer (BCa) is one of the most prevalent urologic malignancies, with a global incidence of approximately 613,000 cases in 2022, ranking it as the ninth most common cancer worldwide^1^. Current epidemiological evidence indicates that cigarette smoking^2^, chronic inflammation^3^, and diabetes mellitus^4^ are significant risk factors for BCa. Epstein-Barr virus (EBV), a widely prevalent human herpesvirus, infects more than 90% of adults worldwide^5^. EBV was the first human oncogenic virus to be discovered^6^, and it has been associated with an increased risk of several cancers, including nasopharyngeal carcinoma^7^, gastric cancer^8^, Hodgkin’s lymphoma^9^, and Burkitt’s lymphoma^10^. Previous observational studies have found an association between EBV infection and the development and progression of BCa ^11–15^. Secreted Frizzled-Related Protein (sFRP) is a class of tumor suppressor genes that blocks the binding of Wnt to its receptor, Frizzled, thereby inhibiting the activation of the Wnt/β-catenin signaling pathway and suppressing cancer development^16^. Many studies have found that low expression of sFRP is associated with the development and progression of BCa^17–21^. Some research has indicated that certain DNA viruses can downregulate sFRP expression through methylation, promoting cancer progression^22–24^. Additionally, it has been shown that methylation of sFRP in BCa inhibits its expression and facilitates the progression of the disease^25^. Furthermore, some studies have indicated that EBV can induce gene methylation^26, 27^. Therefore, we hypothesize that EBV may increase the risk of BCa by suppressing sFRP expression through the induction of methylation. Unfortunately, although observational studies have observed associations between EBV, sFRP, and BCa, these studies are often subject to significant bias due to confounding factors and residual confounding. Moreover, they are unable to establish clear causal relationships between these factors. Therefore, translating findings from observational studies into effective cancer prevention and treatment strategies presents significant challenges.

Mendelian randomization (MR) analysis is an epidemiological research method that uses single nucleotide polymorphisms (SNPs) as instrumental variables (IVs) to assess the causal relationship between exposures (such as viral infections) and diseases (such as cancer). Compared to observational studies MR studies are better able to control for confounding factors, avoid reverse causality, simulate randomized controlled trial designs, and study long-term exposure effects, providing more reliable evidence for causality^28^.

Anti-EBV IgG seropositivity (AEB-IgG), EBV EA-D antibody (EA-D), EBV EBNA-1 antibody (EBNA-1), EBV VCA p18 antibody (VCA-p18), and EBV ZEBRA antibody (ZEBRA) are five commonly used antibodies for assessing EBV infection status in clinical practice and represent readily accessible data for epidemiological studies on EBV infection. Previous studies have found that elevated levels of these EBV infection-related antibodies are associated with the activation of the EBV virus and the progression of cancer^29^. Additionally, other research has shown that increased levels of EBNA antibodies are linked to the recurrence and progression of BCa^15^. And these five antibodies represent different stages of EBV infection and corresponding immune responses. For example, VCA-p18 and EA-D antibodies are associated with early EBV infection, whereas EBNA-1 is associated with latent infection. For this reason, by analysing these antibodies, we were also able to investigate the impact of EBV infection on BCa risk at different stages. To this end, we employed a comprehensive MR approach to investigate the causal relationship between EBV infection-related antibodies (AEB-IgG, EA-D, EBNA-1, VCA-p18, and ZEBRA) and BCa. Furthermore, this study explores whether sFRP mediates these relationships. We hope that this study will contribute to the research on the prevention and treatment of BCa.

## Materials and methods

### Study design

**Fig. 1:**
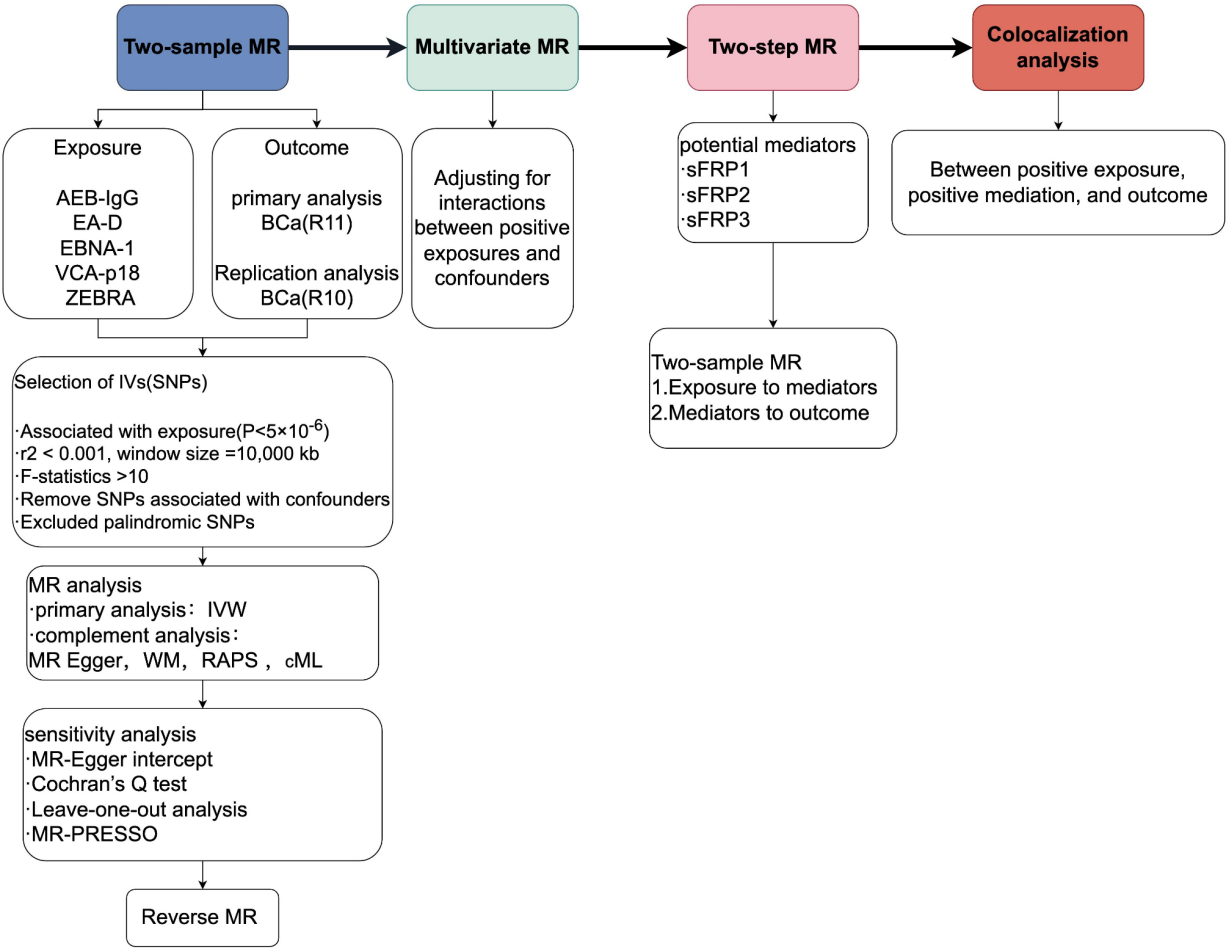
The overview of the study design and flowchart in this study (by Figdraw). SNPs, Single nucleotide polymorphisms; LD, Linkage disequilibrium; MR, Mendelian randomization; IVW, Inverse variance weighted; WM, weighted median; RAPS, Robust adjusted profile score; cML, Constrained maximum likelihood.

MR studies must meet three key core assumptions to ensure its validity^30^ (1) the genetic variant IVs used for MR analysis, must be significantly correlated with the exposure factors studied; (2) the IVs must be independent of potential confounders; and (3) the effect of the IVs on the outcome must be exclusively through the exposure factors, and not by other pathways to directly affect outcomes. Our MR study design met these three core assumptions and followed the STROBE-MR guidelines (Supplementary STROBE-MR-checklist). We first performed a two-sample MR study to assess the causal relationship between 5 EBV-related antibodies (AEB-IgG, EA-D, EBNA-1, VCA-p18, ZEBRA) and BCa using the Finnish Consortium’s R11 dataset, validated with R10. Reverse MR analysis followed. For significant results, multivariable MR (MVMR) was applied to adjust for confounding risk factors. A two-step MR explored the potential mediating role of 3 sFRPs (sFRP1, sFRP2, sFRP3) between positive exposures and BCa. Colocalization analysis were conducted for positive exposures, mediators, and BCa, with multiple sensitivity analyses confirming the robustness of the results. The entire study design is shown in Fig. 1.

### Data sources

All data used in the MR analysis were obtained from publicly available genome-wide association studies (GWAS). GWAS data for five EBV infection-associated antibodies (AEB-IgG, EA-D, EBNA-1, VCA-p18, ZEBRA) were obtained from a UK Biobank (UKB) cohort study containing serologic measurements of up to 10,000 cases of infectious diseases and genome-wide genotyping^31^. We obtained BCa R11 and R10 versions of the GWAS summary data from the FinnGen Consortium (https://finngen.gitbook.io/documentation/). BCa eligible for ICD-O-3 diagnosis with control exclusion for all cancers. GWAS data for smoking (ID:ebi-a-GCST009968), cystitis (ID:ebi-a-GCST90013953), type 2 diabetes (ID:ebi-a-GCST90093110), and three potential mediators of sFRP1 (ID:prot-a-2709), sFRP2 (ID:prot-a-2710), and sFRP3 (ID:prot-a-1140) as risk factors for BCa were obtained from the IEU Open GWAS database (https://gwas.mrcieu.ac.uk/). For more information on the above data, please refer to Supplementary Table 1.

### Instrumental variable selection

We used single nucleotide polymorphisms (SNPs) as IVs for genetic variation. The traditional threshold for selecting genome-wide significant SNPs is *p* < 5 × 10^−^⁸. However, this strict criterion can be problematic if an insufficient number of SNPs are identified, potentially reducing the study’s statistical power or, in some cases, leading to exaggerated results^32^. To mitigate this, we adopted a slightly more lenient p-value threshold when necessary, ensuring a minimum of three SNPs met the criteria for IVs to enhance the power to detect significant associations. First, we extracted SNPs with genome-wide significance for exposure in GWAS, for which we chose a threshold of *p* < 5 × 10^−6^ in the two-sample Mendelian analysis and *p* < 5 × 10^−8^ in the two-step Mendelian randomization analysis. Second, we excluded SNPs with linkage disequilibrium (LD) (r2 < 0.001, clumping distance = 10,000 kb) to eliminated highly associated SNPs. Third, we harmonize the effect sizes and alleles of SNPs in the exposure and outcome data. To prevent weak instrumental variable bias, SNPs with F-statistics < 10 were removed (F = R^2^ (n-k-1)/k(1-R^2^)). To minimize the bias caused by confounding factors, we screened for SNPs strongly associated (*p* < 5 × 10^−8^) with three confounding factors, smoking, cystitis, and type 2 diabetes, by catalog website (https://www.ebi.ac.uk/gwas/). All removed SNPs associated with confounders were shown in Supplementary Table 3. Finally, we used SNPs that met all the above criteria as IVs for the MR analysis. The characteristics of the SNPs used in this study are presented in Supplementary Table 2.

### Statistical analysis

We used the inverse-variance weighted (IVW) method as the primary MR analysis. IVW assumes that all genetic instruments are valid and provides a weighted average of the SNP-specific causal estimates. This method offers high statistical power under the assumption that there is no horizontal pleiotropy^33^. Cochrane’s Q-tests were performed to scrutinize SNP-related heterogeneity for each exposure. In the presence of significant heterogeneity (*p* < 0.05), a random-effects IVW (RE-IVW) model was used; conversely, a fixed-effects IVW (FE-IVW) model was used. In addition, we performed a variety of other complementary MR Methods, including MR-Egger, weighted median (WM), Constrained maximum likelihood (cML), and Robust adjusted profile score (RAPS) to bolster the robustness and credibility of the MR outcomes. MR-Egger can detect some violations of the standard instrumental variable assumptions, and provide an effect estimate which is not subject to these violations. The approach provides a sensitivity analysis for the robustness of the findings from a Mendelian randomization investigation^34^. The MR-Egger method uses the regression intercept as an indicator to test potential multiple effects, and a P value less than 0.05 indicates pleiotropic effects^35^. WM is a method that prevents too many invalid IVs from affecting MR results. This approach can provide a consistent estimate of the causal effect even when up to 50% of the information contributing to the analysis comes from genetic variants that are invalid IVs^36^. Compared to MR-Egger, cML not only eliminates bias caused by uncorrelated pleiotropy but also addresses bias arising from correlated pleiotropy^37^. Due to methodological limitations, it is challenging to completely eliminate the presence of weak instrumental variables, which can introduce bias into the results of MR studies. MR-RAPS takes into account specific pleiotropy and provides robust inferences for MR analyses involving many weak instrumental variables, thereby addressing the biases associated with weak instruments that are inherent in traditional MR studies^38^. The leave-one-out method was used in sensitivity analysis to assess the effect of individual SNPs on overall causal estimates. We also applied MR-PRESSO to detect outliers and found that the removal of outliers effectively reduced horizontal pleiotropy. In addition, we performed sensitivity analysis on the MR results using scatter plots and funnel plots. We used the Steiger Test to detected the selected SNPs for potential reverse causality between EBV-related antibodies (AEB-IgG, EA-D, EBNA-1, VCA-p18, ZEBRA) and BCa (Supplementary Table 7). To control the false-positive error rate, multiple comparisons in the MR study were adjusted using false discovery rate (FDR) correction. A P_fdr < 0.05 was considered statistically significant, indicating a robust causal relationship between the exposure and the outcome. However, when *P* < 0.05 but P_fdr > 0.05, the result was interpreted as suggesting a potential causal relationship. We performed reverse MR using the same criteria and also performed two-sample MR as a repeat validation using the Finnish R10 version of BCa data as the outcome. For causally related exposures and outcomes, we confirmed direct causality by adjusting for the interaction of relevant risk factors and exposures using multivariate MR(MVMR).

To investigate the potential causal mechanisms between EBV infection and BCa, we conducted a two-step MR mediation analysis. Previous studies have shown that abnormal expression of sFRP is associated with BCa^39^, and certain DNA viruses have been found to downregulate sFRP expression, promoting cancer progression^22–24^. Thus, we selected sFRP proteins (sFRP1, sFRP2, sFRP3) as potential mediators. First, we applied two-sample MR to examine the causal effect of positive exposure (VCA p18) on the potential mediators. Positive results were used as possible potential positive mediators. We then proceeded to assess the causal relationship between potentially positive mediators and BCa using two-sample MR (SNPs associated with exposure were excluded from further analysis). Additionally, we performed colocalization analysis on exposures, potential mediators, and outcomes that showed causal relationships in the MR study to determine whether they share the same causal variants^40^. Within a Bayesian framework, we calculated posterior probabilities for five hypotheses (H0-H4). A posterior probability of H4 (PP.H4) ≥ 80% in the colocalization analysis indicates a significant shared causal variant, while 50% < PP.H4 < 80% suggests a potential shared causal variant^41^.

All statistical analysis and data visualizations were performed with the “TwoSampleMR”, “MRPRESSO”, “Forestplot”, “coloc”, “MRcML” R packages in R software version 4.3.3 (R Foundation for Statistical Computing, Vienna, Austria).

## Results

### Two-sample MR

**Fig. 2:**
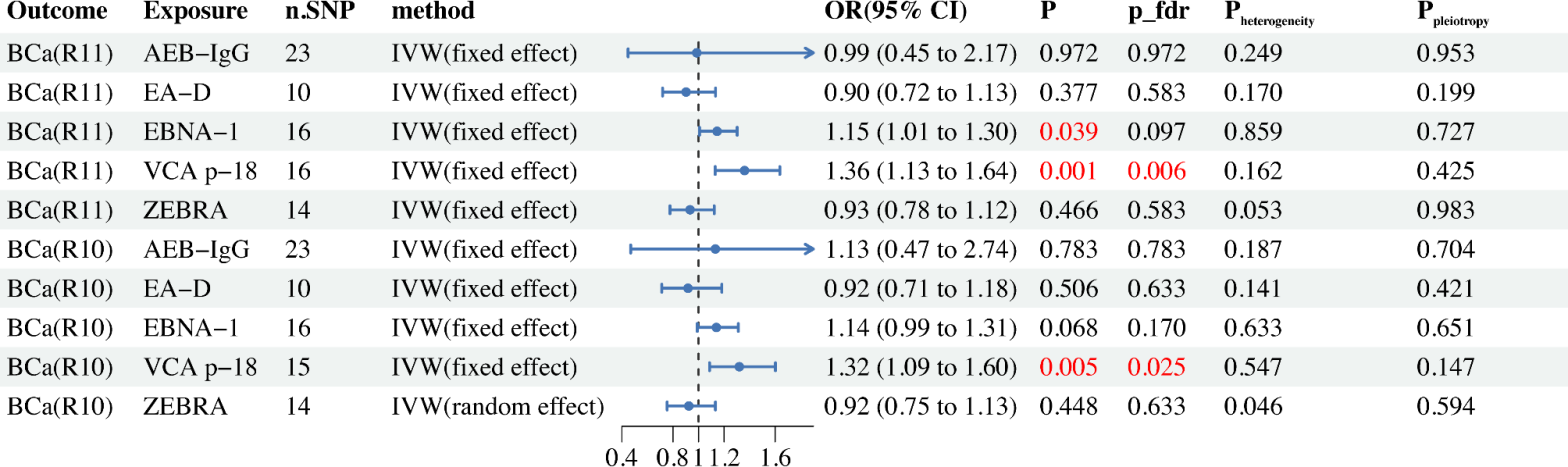
Two-sample MR forest plots for primary analysis and validation analysis.

In the primary analysis of 5 antibodies associated with EBV infection and BCa (R11), we screened a total of 79 eligible SNPs for two-sample MR. Details of the selected SNPs are shown in Supplementary Table 2. The results of the MR analysis showed that EBNA-1 may increase BCa risk. Through Cochran’s Q test (Supplementary Table 6), we did not find heterogeneity in gene IVS related to EBNA-1 (P_heterogeneity_ = 0.859), so we selected the FE-IVW as the main MR analysis. FE-IVW results showed that EBNA-1 increased the risk of BCa (OR = 1.15, 95% CI 1.01–1.30; *p* = 0.039) (Fig. 2). RAPS (OR = 1.15, 95% CI 1.01–1.32; *p* = 0.040) methods also yielded results consistent with FE-IVW. However, MR-Egger (OR = 1.10, 95% CI 0.85–1.42; *p* = 0.471), WM (OR = 1.09, 95% CI 0.91–1.29; *p* = 0.344), cML (OR = 1.15, 95% CI 0.99–1.33; *p* = 0.059) results showed no statistical significance, but exhibited the same trend. Our MR study found that VCA-p18 significantly increased the risk of BCa (Fig. 2). As shown in Supplementary Table 6, we chose FE-IVW as the primary MR analysis because its Cochran’s Q test showed no heterogeneity (P_heterogeneity_ = 0.162). FE-IVW analysis found that VCA-p18 (OR = 1.36, 95% CI 1.13–1.64; *p* = 0.001) significantly increased the risk of BCa, and three methods, WM (OR = 1.44, 95% CI 1.14–1.83; *p* = 0.003), cML (OR = 1.38, 95% CI 1.09–1.75; *p* = 0.007), and RAPS (OR = 1.38, 95% CI 1.12–1.69; *p* = 0.003), yielded results consistent with FE-IVW. Although MR-Egger (OR = 1.16, 95% CI 0.76–1.77; *p* = 0.499) results showed no statistical significance, a direction of effect consistent with other methods was obtained. After FDR correction (P_fdr = 0.006), VCA-p18 remained a significant risk factor for BCa. In sensitivity analyses, neither the MR-Egger intercept nor the MR-PRESSO test indicated evidence of pleiotropy (Supplementary Table 5). Additionally, the leave-one-out analysis did not identify any SNPs that introduced significant bias to the results. Funnel plots and forest plots are provided in Supplementary Fig. 1. These sensitivity analyses collectively support the robustness of the findings.

In the replication MR analysis using the Finnish R10 version of BCa data, we found that only VCA-p18 significantly increased the risk of BCa (OR = 1.32, 95% CI 1.09–1.60; *p* = 0.005). Similarly, after FDR correction (P_fdr = 0.025), VCA p18 remained significantly associated with an elevated risk of BCa. Sensitivity (Supplementary Table 5) and heterogeneity (Supplementary Table 6) analyses indicated no issues, supporting the robustness of these findings. The replication results align closely with those of the primary analysis using the Finnish R11 version, further confirming the reliability of our results.

We performed reverse MR analysis using SNP selection thresholds consistent with the main analysis. We did not find an inverse causal relationship between BCa and antibodies associated with EBV infection. Detailed results are presented in (Supplementary Table 4).

### MVMR

**Fig. 3:**
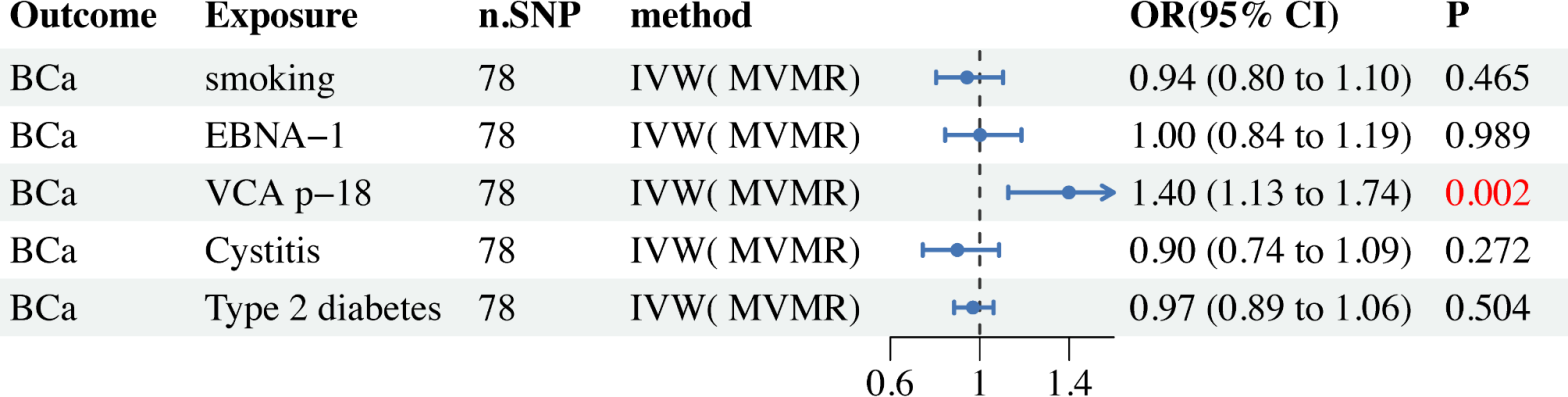
MVMR forest plot of VCA-P18 and EBNA-1 with bladder cancer risk factors (smoking, cystitis, type 2 diabetes).

To mitigate the mutual influence between positive exposure and confounding factors (smoking, cystitis, type 2 diabetes), we conducted MVMR to explore their direct causal relationships with BCa. After adjusting for mutual effects in MVMR, the results indicated that the effect of VCA-p18 on BCa risk was enhanced, whereas the effect of EBNA-1 on BCa disappeared (Fig. 3). The MVMR Egger intercept test showed no evidence of horizontal pleiotropy (P_pleiotropy_ = 0.964). These MVMR findings further support that VCA-p18 increases the risk of BCa.

### Two-step MR

**Fig. 4:**
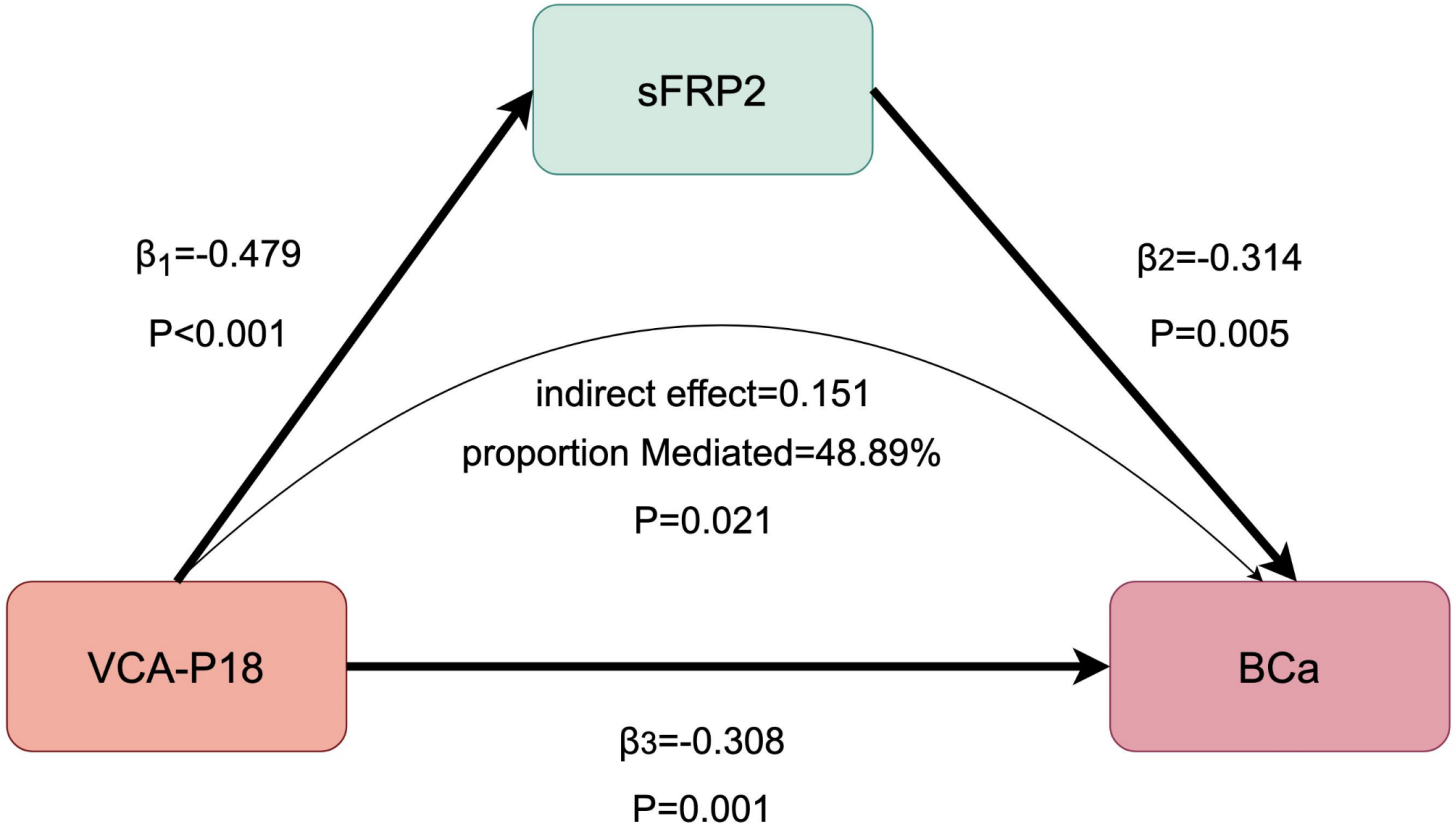
Schematic diagram of mediating effects of sFRP2 levels. β1 represents the causal effect of Epstein-Barr virus VCA p18 antibody levels (VCA-P18) on Secreted frizzled-related protein 2(sFRP2); β2 represents the causal effect of sFRP2 on bladder cancer (BCa); β3 represents the total causal effect of VCA-P18 on BCa. Indirect effect = β1 × β2. Proportion mediated was the indirect effect divided by the total effect (Proportion mediated = β1 × β2/β3).

Our two-sample MR analysis with positive exposure to VCA-p18 with potential mediator sFRPs (sFRP1, sFRP2, sFRP3) revealed a significant causal relationship between VCA-p18 and sFRP2 (Beta = − 0.479, 95% CI − 0.709 to − 0.250, *P* = 4.12E−05), and the detailed results are presented in the Supplementary Table 8. We further explored the causal relationship between the potential positive mediator sFRP2 and BCa using two-sample MR and found that sFRP2 significantly reduced the risk of BCa (Beta = − 0.314, 95% CI − 0.533 to − 0.095, *P* = 0.005). The final two-step Mendelian randomisation showed that VCA-p18 reduced sFRP2 expression increasing the risk of BCa (Fig. 4).

### Colocalization analysis

**Fig. 5:**
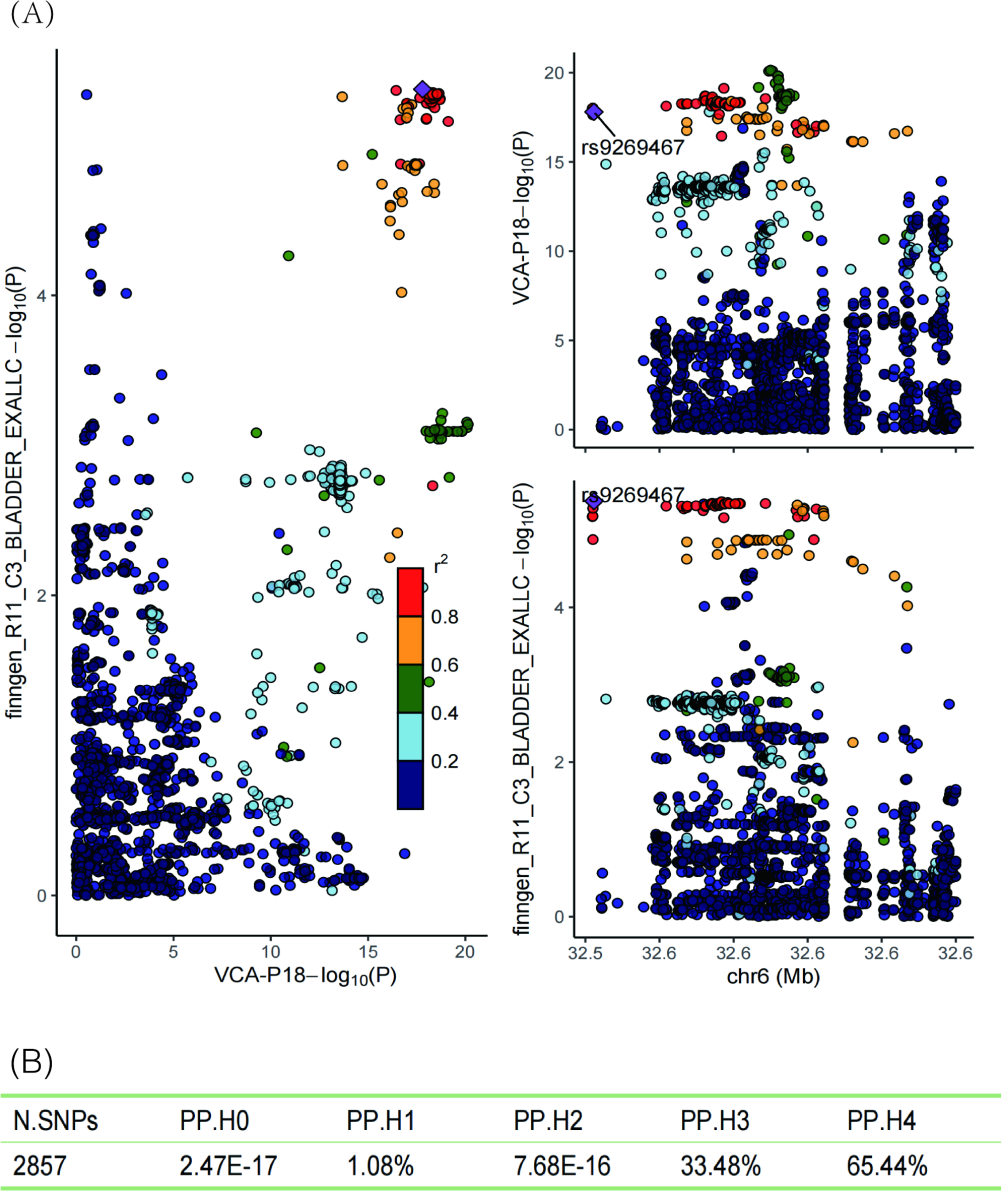
(**A**) Regional association plots for VCA-P18 and bladder cancer (BCa) at the chromosome 6 locus overlapping HLA-DQA1. (**B**) Colocalization analysis of VCA-P18 and BCa. PP.H0 = neither VCA-P18 nor BCa risk has a genetic association in the region, PP.H1 = only VCA-P18 has a genetic association in the region, PP.H2 = only BCa risk has a genetic association in the region, PP.H3 = both VCA-P18 and BCa risk are associated but have different causal variants, PP.H4 = both VCA-P18 and BCa risk are associated and share a single causal variant.

In the colocalization analysis among VCA-p18, sFRP2, and BCa, we identified a potential shared causal variant only between VCA-p18 and BCa (PP.H4 = 65.44%), further supporting the causal effect of VCA-p18 on BCa (Supplementary Table 9). Additionally, the colocalization analysis revealed that the lead SNP shared between VCA-p18 and BCa is rs9269467, located near the HLA-DQA1 gene (Fig. 5).

## Discussion

EBV, also known as human herpesvirus 4, is a DNA virus within the herpesvirus family and one of the most widely distributed viruses globally^42^. EBV infection is generally asymptomatic or causes mild symptoms, though in some individuals it can lead to infectious mononucleosis, characterized by fever, sore throat, lymphadenopathy, and fatigue. Although previously considered largely harmless, emerging data clearly underscore the pathological role of lifelong EBV infection in driving autoimmunity and malignancy in a small yet significant subset of the population^43^. Since its discovery in 1964 as the first human oncogenic virus^6^, EBV infection has been linked to an increased risk of several cancers, accounting for approximately 1.5% of all human cancer cases worldwide^44^. Its associations with nasopharyngeal carcinoma^7^, gastric cancer^8^, and Hodgkin’s lymphoma^9^ are particularly well-established. The discovery of EBV laid the foundation for subsequent research on human oncogenic viruses, providing crucial insights into cancer prevention, diagnosis, and treatment. Observational studies have suggested an association between EBV infection and the incidence of BCa, yet the causal relationship remains uncertain^11–15^. This study employs two-sample MR to provide genetic evidence that EBV infection increases the risk of BCa. Specifically, our findings present strong evidence that EBV infection (VCA-p18) can increase BCa risk by downregulating sFRP2 levels. Additionally, EBNA-1 antibodies may also contribute to an elevated risk of BCa.

The VCA-p18 antibody is a specific antibody produced against the p18 protein within the EBV viral capsid antigen (VCA). Detection of VCA-p18 antibodies is primarily used to determine whether an individual has been previously infected with EBV or is experiencing an active EBV infection. Upon initial EBV infection, VCA-p18 IgM antibodies typically appear in the early stages, indicating an acute infection. As the infection progresses, VCA-p18 IgG antibodies are generated and persist long-term, suggesting past or chronic infection^43^. The presence of VCA-p18 antibodies often corresponds with the lytic phase of EBV infection, during which the virus actively replicates. This phase triggers a chronic inflammatory response, marked by the release of various pro-inflammatory factors (e.g., IL-6 and TNF-α)^42^. Chronic inflammation is considered a key factor in tumorigenesis, potentially promoting tumor formation by driving cell proliferation and inducing DNA damage^45^. In addition it was found that binding of VCA-p18 antibody to FcγRIIIB could drive antibody-dependent neutrophil phagocytosis during both acute and chronic infection phases^43, 46^. Prolonged neutrophil activation may induce chronic inflammatory responses and oxidative stress in local tissues, thereby increasing the risk of cellular mutations and carcinogenesis. Our study also found that HLA-DQA1 gene variants may be an important reason for the increased risk of BCa associated with VCA-p18. The HLA-DQA1 gene belongs to the HLA class II genes, encoding molecules that play a key role in antigen presentation by presenting exogenous antigens (such as viral proteins) to CD4+ or CD8+ T cells, thereby triggering a specific immune response^47^. Variations in the HLA-DQA1 gene have been found to be associated with the development of several cancers^48–51^. If specific variations occur in the HLA-DQA1 gene, leading to reduced binding and presentation efficiency of the VCA-p18 antigen, T cell activation may be insufficient, weakening the ability to clear infected cells. This inefficient immune response may facilitate immune evasion by EBV, allowing infected cells to persist and proliferate, thereby increasing the risk of BCa development. Overall, the reasons for the increased risk of BCa with VCA p-18 antibodies may be related to the chronic inflammation and abnormalities in the immune system response that they cause.

sFRPs are a class of important signaling molecules that belong to the Frizzled receptor family. They are a group of secreted proteins that inhibit the Wnt signaling pathway. The Wnt signaling pathway plays a critical role in cell development, proliferation, differentiation, and tissue homeostasis, and sFRPs can regulate this pathway through their interaction with Wnt proteins^16, 52^. Aberrant activation of the Wnt signaling pathway is often associated with the occurrence and progression of various cancers; thus, dysfunction of sFRPs may contribute to cancer development^16^. Previous studies have found that abnormal expression of sFRPs is related to the occurrence and progression of BCa. In BCa, previous studies have found that hypermethylation reduces sFRP1 expression, promoting BCa development through regulation of the canonical Wnt pathway^18, 21, 25^, as well as the non-canonical Wnt and MAPK pathways^20^. Furthermore, although we did not find GWAS data for sFRP4 and were unable to conduct MR analysis to explore the relationship between sFRP4 and BCa, as well as its potential mediating role in the relationship between EBV and BCa, previous studies have indicated that its abnormal expression is also associated with the occurrence and progression of BCa^17^. Additionally, studies have found that low expression of sFRP2 in BCa tumor tissues is associated with poor prognosis^19^. Previous studies have found that viral infections can suppress the expression of sFRPs, thereby promoting the development of cancer^23, 53^. Our research using MR analysis revealed that infection with EBV, indicated by VCA-p18 antibodies, increases the risk of BCa. We hypothesize that EBV may also elevate the risk of BCa by inhibiting the expression of sFRPs. To investigate this, we conducted a two-step MR mediation analysis to explore whether sFRPs (sFRP1, sFRP2, sFRP3) mediate the relationship between VCA-p18 antibodies and BCa risk. The results of the two-step MR analysis indicated that VCA-p18 downregulates sFRP2, thereby increasing the risk of BCa. In BCa, studies have found that sFRP2 not only promotes apoptosis and reduces proliferation rates through the Wnt signaling pathway or other pathways regulating cell survival, but it also stabilizes the extracellular matrix, thereby limiting the invasive behavior of tumor cells^19^. Therefore, its reduction may increase the risk of BCa. Epigenetic alterations of the sFRP gene, particularly hypermethylation of the gene promoter, are one of the main causes of its reduced expression. In various cancers, such as colorectal cancer^54^ and gastric cancer^55^, abnormal methylation of the sFRP2 promoter region leads to transcriptional suppression, thereby decreasing gene expression. Some studies have found that infections with certain DNA viruses, such as hepatitis B virus^56^, hepatitis C virus^24^, and human papillomavirus^22, 23^, can increase methylation of sFRP genes. Although no studies have specifically found that EBV increases sFRP2 methylation, it has been shown that EBV has the capability to enhance gene methylation. Research has shown that the EBV viral latent membrane protein 2A can induce the phosphorylation of STAT3, leading to the upregulation of DNMT1 expression and subsequently inducing gene methylation^27^. Therefore, we speculate that EBV-induced sFRP2 methylation may be one of the reasons for the downregulation of sFRP2 expression by EBV. The potential oncogenic protein LMP1 encoded by EBV can activate multiple signaling pathways in host cells, particularly pro-inflammatory and proliferative pathways such as NF-κB and AP-1, which may also inhibit the expression of the Wnt antagonist sFRP2^26^. Additionally, EBV infection is often accompanied by chronic inflammation, leading to the release of various immune modulators and pro-inflammatory factors, which may indirectly suppress sFRP2 expression^42^. These may be the reasons why VCA-p18 downregulates sFRP2 and thus increases BCa risk.

Epstein-Barr virus EBNA-1 antibodies are specific antibodies targeting Epstein-Barr nuclear antigen 1of EBV. EBNA-1 is a key protein that is persistently expressed during latent EBV infection, primarily functioning to maintain the viral genome within host cells^43, 57^. Our study found that EBNA-1 antibodies may also be associated with an increased risk of BCa. Although we did not find studies directly linking EBNA-1 to BCa, some studies suggest that EBNA-1 has oncogenic potential. Wilson et al.^58^ used a transgenic mouse model in which EBNA-1 was expressed under the control of the mouse Ig heavy chain intronic enhancer, and their experimental results demonstrated that EBNA-1 is oncogenic in vivo and may directly participate in the mechanisms underlying the development of Burkitt’s lymphoma and other EBV-associated malignancies. Lu et al.^59^ suggested that EBNA-1 promotes carcinogenesis by upregulating the apoptosis inhibitor survivin in EBV-associated B lymphoma cells. Additionally, EBNA-1 protein facilitates the stable attachment of the EBV genome to host cell chromosomes, ensuring the persistent transmission of the virus during cell division^42^. This continuous infection may also increase genomic instability in the host cell and elevate the risk of cancer.

The strength of our study lies in the comprehensive and systematic evaluation of EBV infection and BCa risk, as well as the exploration of the potential mediating role of sFRP and the possible mechanisms underlying these relationships. However, this study also has several limitations. First, the study population was limited to individuals of European ancestry, which may restrict the generalizability of the findings to other populations. Second, although we employed various sensitivity tests to validate our results, we could not test the independence assumption or exclusion restriction in MR analysis, so the possibility of pleiotropic effects cannot be entirely ruled out. Third, the GWAS data used were summary-level data without individual-specific information, which prevented us from conducting subgroup analyses. Fourth, our analysis focused solely on cancer risk rather than disease progression, which limits our ability to provide insights into the potential utility of targeted biomarkers in cancer treatment. Fifth, due to the inability to find GWAS data for sFRP4 and sFRP5, we were unable to explore whether they mediate the relationship between EBV and BCa. Given these limitations, further experimental and clinical studies are needed to validate these findings and determine their potential value in clinical practice.

## Conclusions

To our knowledge, this is the first study to explore the causal relationship between EBV infection and BCa risk using MR analysis. Our study suggests that the VCA-p18 antibody associated with EBV infection may increase the risk of BCa by lowering sFRP2 levels. Additionally, EBNA-1 antibodies may also contribute to an elevated BCa risk. These findings suggest that chronic EBV infection may be a risk factor for BCa. We hope these results provide new insights for future research on the relationship between EBV and BCa.

## Electronic supplementary material

Below is the link to the electronic supplementary material.

Supplementary Material 1

Supplementary Material 2

Supplementary Material 3

## Data Availability

All data generated or analysed during this study are included in this published article [and its supplementary information files].

## Abbreviations

AEB-IgG: Anti-Epstein-Barr virus IgG seropositivity
BCa: Bladder cancer
cML: Constrained maximum likelihood
EA-D Ab: Epstein-Barr virus EA-D antibody
EBNA-1Ab: Epstein-Barr virus EBNA-1 antibody
EBV: Epstein-Barr virus
FDR: False Discovery Rate
FE-IVW: Fixed-effects Inverse variance weighted
GWAS: Genome-wide association study
IVs: Instrumental variables
IVW: Inverse variance weighted
LD: Linkage disequilibrium
MR: Mendelian randomization
MVMR: Multivariate Mendelian randomization
RAPS: Robust adjusted profile score
RE-IVW: random-effects Inverse variance weighted
sFRP: Secreted frizzled-related protein
SNP: Single nucleotide polymorphism
UKB: UK Biobank
VCA-p18 Ab: Epstein-Barr virus VCA p18 antibody levels
WM: Weighted median
ZEBRA: Epstein-Barr virus ZEBRA antibody levels
