# Supplementary material for: SFRP2 mediates Epstein-Barr virus and bladder cancer risk: a Mendelian randomization study and colocalization analysis
Source: Sci Rep. 2025 Feb 28;15:7118. doi: 10.1038/s41598-025-91594-9 (PMC11868617; doi:10.1038/s41598-025-91594-9)
Supplement: Supplementary file 1 — Supplementary Material 1 [file 41598_2025_91594_MOESM1_ESM.pdf]

STROBE-MR checklist of recommended items to address in reports of Mendelian randomization studies[1 2]

Item No.	Section	Checklist item	Page No.	Relevant text from manuscript
1	**TITLE and ABSTRACT**	Indicate Mendelian randomization (MR) as the study's design in the title and/or the abstract if that is a main purpose of the study	1	Title: sFRP2 mediates Epstein-Barr virus and bladder cancer risk: a Mendelian randomization study and colocalization analysis.
	**INTRODUCTION**			
2	**Background**	Explain the scientific background and rationale for the reported study. What is the exposure? Is a potential causal relationship between exposure and outcome plausible? Justify why MR is a helpful method to address the study question	3-4	Background: The study explores the causal relationships between Epstein-Barr virus (EBV) infection-related antibodies and bladder cancer (BCa) risk, mediated by sFRP2, a tumor suppressor gene. EBV's role as an oncogenic virus is known in other cancers, and this study hypothesizes that EBV may contribute to BCa through sFRP2 modulation.
3	**Objectives**	State specific objectives clearly, including pre-specified causal hypotheses (if any). State that MR is a method that, under specific assumptions, intends to estimate causal effects	4	Objectives: To examine the causal relationship between EBV infection and BCa, and investigate sFRP as a mediator.
	**METHODS**			
4	**Study design and data sources**	Present key elements of the study design early in the article. Consider including a table listing sources of data for all phases of the study. For each data source contributing to the analysis, describe the following:	4-7	Study design: Two-sample Mendelian randomization (MR) and colocalization analysis were used. GWAS data on EBV antibodies and BCa were sourced from UK Biobank and the FinnGen Consortium, respectively. The study population included individuals of European ancestry.
	a)	Setting: Describe the study design and the underlying population, if possible. Describe the setting, locations, and relevant dates, including periods of recruitment, exposure, follow-up, and data collection, when available.		
	b)	Participants: Give the eligibility criteria, and the sources and methods of selection of participants. Report the sample size, and whether any power or sample size calculations were carried out prior to the main analysis		
	c)	Describe measurement, quality control and selection of genetic variants		
	d)	For each exposure, outcome, and other relevant variables, describe methods of assessment and diagnostic criteria for diseases		

		e)	Provide details of ethics committee approval and participant informed consent, if relevant		
5	**Assumptions**		Explicitly state the three core IV assumptions for the main analysis (relevance, independence and exclusion restriction) as well assumptions for any additional or sensitivity analysis	4-5	Assumptions: The study assumes that (1) the genetic instruments (SNPs) are associated with EBV exposure, (2) they are independent of confounders, and (3) they influence BCa risk only through EBV exposure and sFRP2 mediation.
6	**Statistical methods: main analysis**		Describe statistical methods and statistics used	6-7	Statistical methods: Primary analysis used inverse-variance weighted (IVW) MR, with additional MR methods (MR-Egger, weighted median, RAPS, and MR-PRESSO) to validate results. Sensitivity analyses included heterogeneity tests and pleiotropy assessment.
		a)	Describe how quantitative variables were handled in the analyses (i.e., scale, units, model)		
		b)	Describe how genetic variants were handled in the analyses and, if applicable, how their weights were selected		
		c)	Describe the MR estimator (e.g. two-stage least squares, Wald ratio) and related statistics. Detail the included covariates and, in case of two-sample MR, whether the same covariate set was used for adjustment in the two samples		
		d)	Explain how missing data were addressed		
		e)	If applicable, indicate how multiple testing was addressed		
7	**Assessment of assumptions**		Describe any methods or prior knowledge used to assess the assumptions or justify their validity	6-7	Assessment of assumptions: We used Cochrane's Q-test to check for SNP-related heterogeneity. MR-Egger intercept test was used to detect potential directional pleiotropy. Leave-one-out analysis was conducted to assess the influence of individual SNPs. The MR-PRESSO method was used to detect outlier SNPs.
8	**Sensitivity analyses and additional analyses**		Describe any sensitivity analyses or additional analyses performed (e.g. comparison of effect estimates from different approaches, independent replication, bias analytic techniques, validation of instruments, simulations)	7-8	We performed sensitivity analyses using different MR methods (e.g., MR-Egger, WM, cML, dIVW, RAPS, BWMR). We also conducted colocalization analysis to determine whether the observed associations shared a common causal variant.
9	**Software and pre-registration**			8	All statistical analyses and data visualizations were performed using the “TwoSampleMR,” “MRPRESSO,” “coloc,” ,“Forestplot,” and “MRcML” R packages in R software version 4.3.3.

		a) Name statistical software and package(s), including version and settings used		
		b) State whether the study protocol and details were pre-registered (as well as when and where)		
	**RESULTS**			
10	**Descriptive data**		5	Descriptive data: Data for EBV antibody levels and BCa risk were derived from large GWAS datasets (IEU OPEN and FinnGen) with high sample sizes. No individual-level data analysis was performed.
		a) Report the numbers of individuals at each stage of included studies and reasons for exclusion. Consider use of a flow diagram		
		b) Report summary statistics for phenotypic exposure(s), outcome(s), and other relevant variables (e.g. means, SDs, proportions)		
		c) If the data sources include meta-analyses of previous studies, provide the assessments of heterogeneity across these studies		
		d) For two-sample MR:<br>i. Provide justification of the similarity of the genetic variant-exposure associations between the exposure and outcome samples<br>ii. Provide information on the number of individuals who overlap between the exposure and outcome studies		
11	**Main results**			
		a) Report the associations between genetic variant and exposure, and between genetic variant and outcome, preferably on an interpretable scale	8-10	MR results indicated that VCA-p18 antibody significantly increased BCa risk (OR = 1.36, 95% CI: 1.13-1.64, p=0.001). sFRP2 was identified as a mediator between VCA-p18 and BCa.
		b) Report MR estimates of the relationship between exposure and outcome, and the measures of uncertainty from the MR analysis, on an interpretable scale, such as odds ratio or relative risk per SD difference		
		c) If relevant, consider translating estimates of relative risk into absolute risk for a meaningful time period		
		d) Consider plots to visualize results (e.g. forest plot, scatterplot of associations between genetic variants and outcome versus between genetic variants and exposure)		
12	**Assessment of assumptions**		8	The Cochrane's Q-test revealed no significant heterogeneity for some BUB, supporting the validity of the MR analysis. The MR-Egger intercept test indicated no evidence of directional pleiotropy.

					Leave-one-out analysis and MR-PRESSO tests confirmed the robustness of the results.
		a)	Report the assessment of the validity of the assumptions		
		b)	Report any additional statistics (e.g., assessments of heterogeneity across genetic variants, such as I^2, Q statistic or E-value)		
13	**Sensitivity analyses and additional analyses**			9	Sensitivity analyses: Bidirectional MR and colocalization analysis confirmed causal relationships. Reverse MR ruled out reverse causality.
		a)	Report any sensitivity analyses to assess the robustness of the main results to violations of the assumptions		
		b)	Report results from other sensitivity analyses or additional analyses		
		c)	Report any assessment of direction of causal relationship (e.g., bidirectional MR)		
		d)	When relevant, report and compare with estimates from non-MR analyses		
		e)	Consider additional plots to visualize results (e.g., leave-one-out analyses)		
	**DISCUSSION**				
14	**Key results**		Summarize key results with reference to study objectives	10	Significant associations between EBV infection (VCA-p18 antibody) and BCa risk were identified, with sFRP2 as a mediator.
15	**Limitations**		Discuss limitations of the study, taking into account the validity of the IV assumptions, other sources of potential bias, and imprecision. Discuss both direction and magnitude of any potential bias and any efforts to address them	13	The study population was limited to individuals of European ancestry, potentially limiting the generalizability of the findings. The possibility of multiple effects cannot be completely ruled out despite various sensitivity tests. The analysis was also limited to cancer risk rather than progression, and thus does not provide information on the utility of targeted biomarkers in cancer treatment.
16	**Interpretation**				
		a)	Meaning: Give a cautious overall interpretation of results in the context of their limitations and in comparison with other studies	10-13	EBV, specifically through VCA-p18 antibodies, may increase BCa risk via downregulation of sFRP2. These findings suggest potential targets for BCa prevention in individuals with EBV infection.
		b)	Mechanism: Discuss underlying biological mechanisms that could drive a potential causal relationship between the investigated exposure and the outcome, and whether the gene-environment equivalence assumption is reasonable. Use causal language carefully, clarifying that IV estimates may provide causal effects only under certain		

		assumptions		
		c) Clinical relevance: Discuss whether the results have clinical or public policy relevance, and to what extent they inform effect sizes of possible interventions		
17	**Generalizability**	Discuss the generalizability of the study results (a) to other populations, (b) across other exposure periods/timings, and (c) across other levels of exposure	13	The study's findings are primarily applicable to individuals of European ancestry, and further research is needed to determine their relevance to other populations and settings.
	**OTHER INFORMATION**			
18	**Funding**	Describe sources of funding and the role of funders in the present study and, if applicable, sources of funding for the databases and original study or studies on which the present study is based	20	This study was supported by grants from the National Natural Science Foundation of China (NO. 82160927, 82274610, 82260957) and other sources
19	**Data and data sharing**	Provide the data used to perform all analyses or report where and how the data can be accessed, and reference these sources in the article. Provide the statistical code needed to reproduce the results in the article, or report whether the code is publicly accessible and if so, where	20	We acknowledge the IEU Open GWAS and FinnGen consortia for providing GWAS data.
20	**Conflicts of Interest**	All authors should declare all potential conflicts of interest	20	The authors declare no conflicts of interest.

This checklist is copyrighted by the Equator Network under the Creative Commons Attribution 3.0 Unported (CC BY 3.0) license.

1. Skrivankova VW, Richmond RC, Woolf BAR, Yarmolinsky J, Davies NM, Swanson SA, et al. Strengthening the Reporting of Observational Studies in Epidemiology using Mendelian Randomization (STROBE-MR) Statement. JAMA. 2021;under review.

2. Skrivankova VW, Richmond RC, Woolf BAR, Davies NM, Swanson SA, VanderWeele TJ, et al. Strengthening the Reporting of Observational Studies in Epidemiology using Mendelian Randomisation (STROBE-MR): Explanation and Elaboration. BMJ. 2021;375:n2233.
