# Supplementary material for: SFRP2 mediates Epstein-Barr virus and bladder cancer risk: a Mendelian randomization study and colocalization analysis
Source: Sci Rep. 2025 Feb 28;15:7118. doi: 10.1038/s41598-025-91594-9 (PMC11868617; doi:10.1038/s41598-025-91594-9)

Scatterplot of BCa（R11）

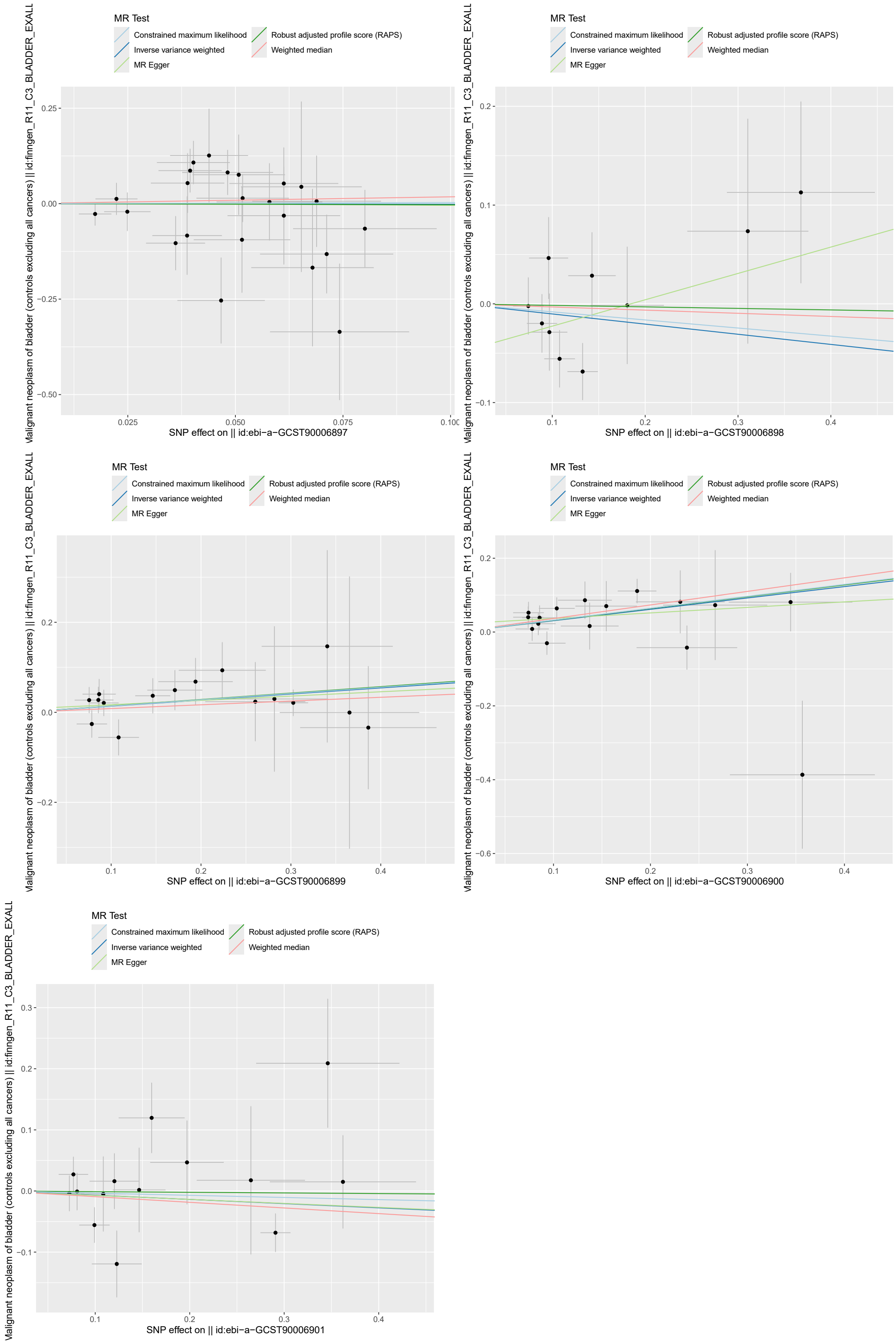

Forest map of BCa（R11）

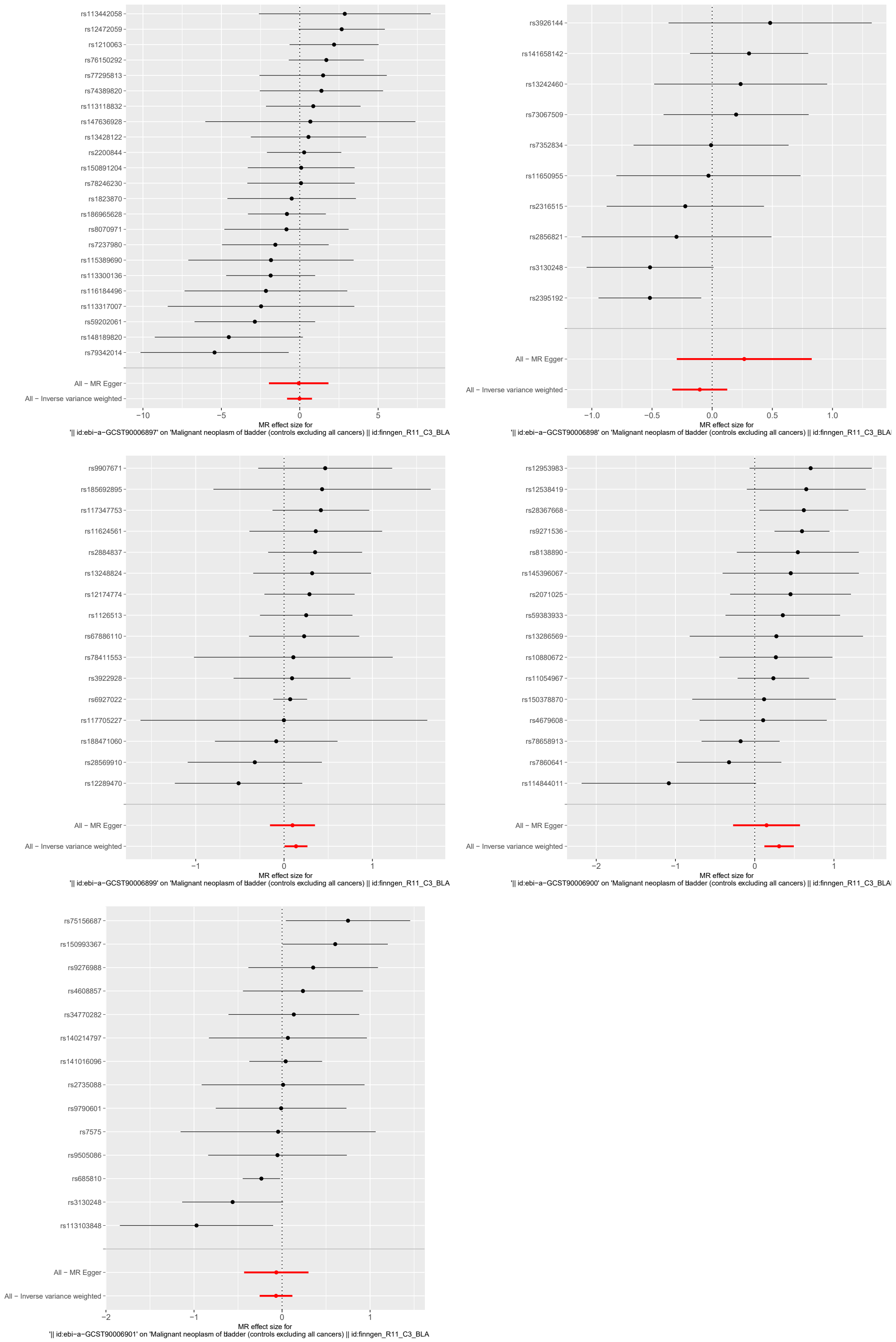

funnel plot of BCa（R11）

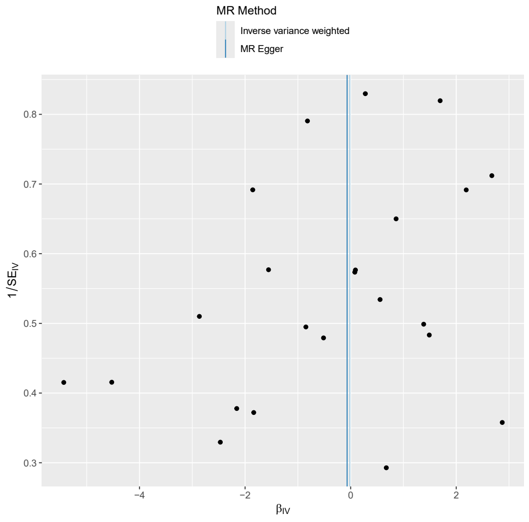

AEB-IgG

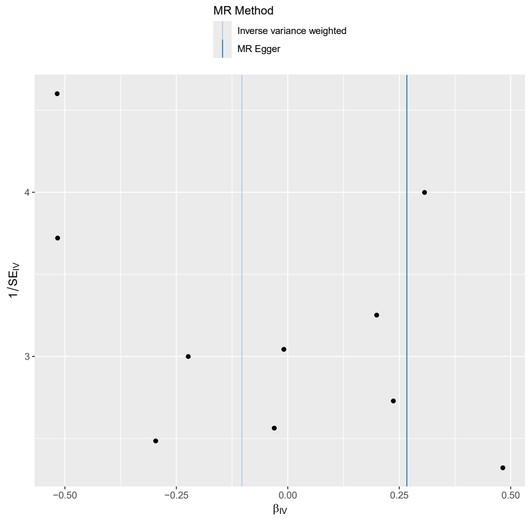

EA-D

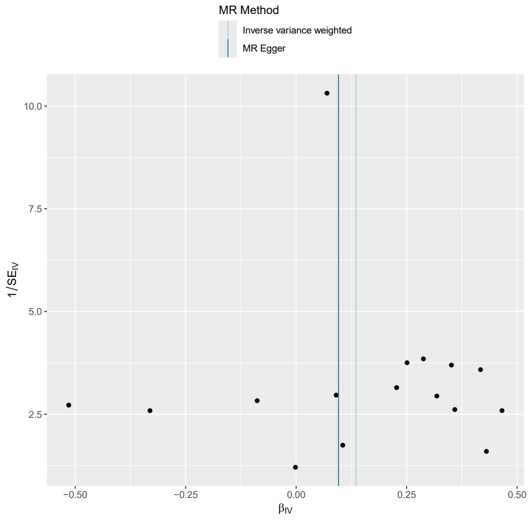

EBNA-1

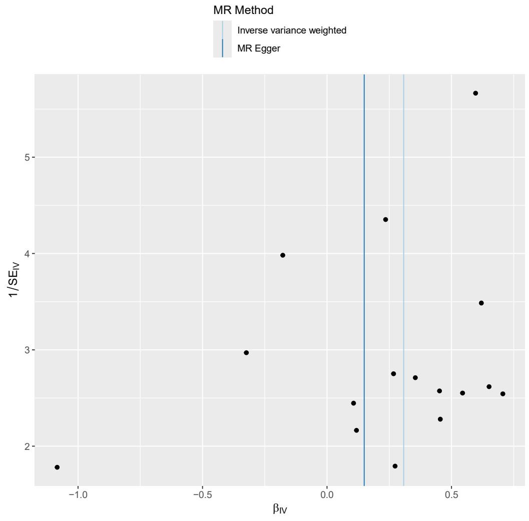

VCA-p18

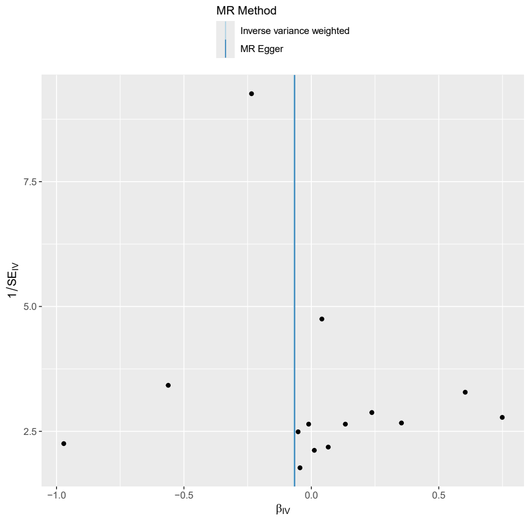

ZEBRA

leave-one-out of BCa（R11）

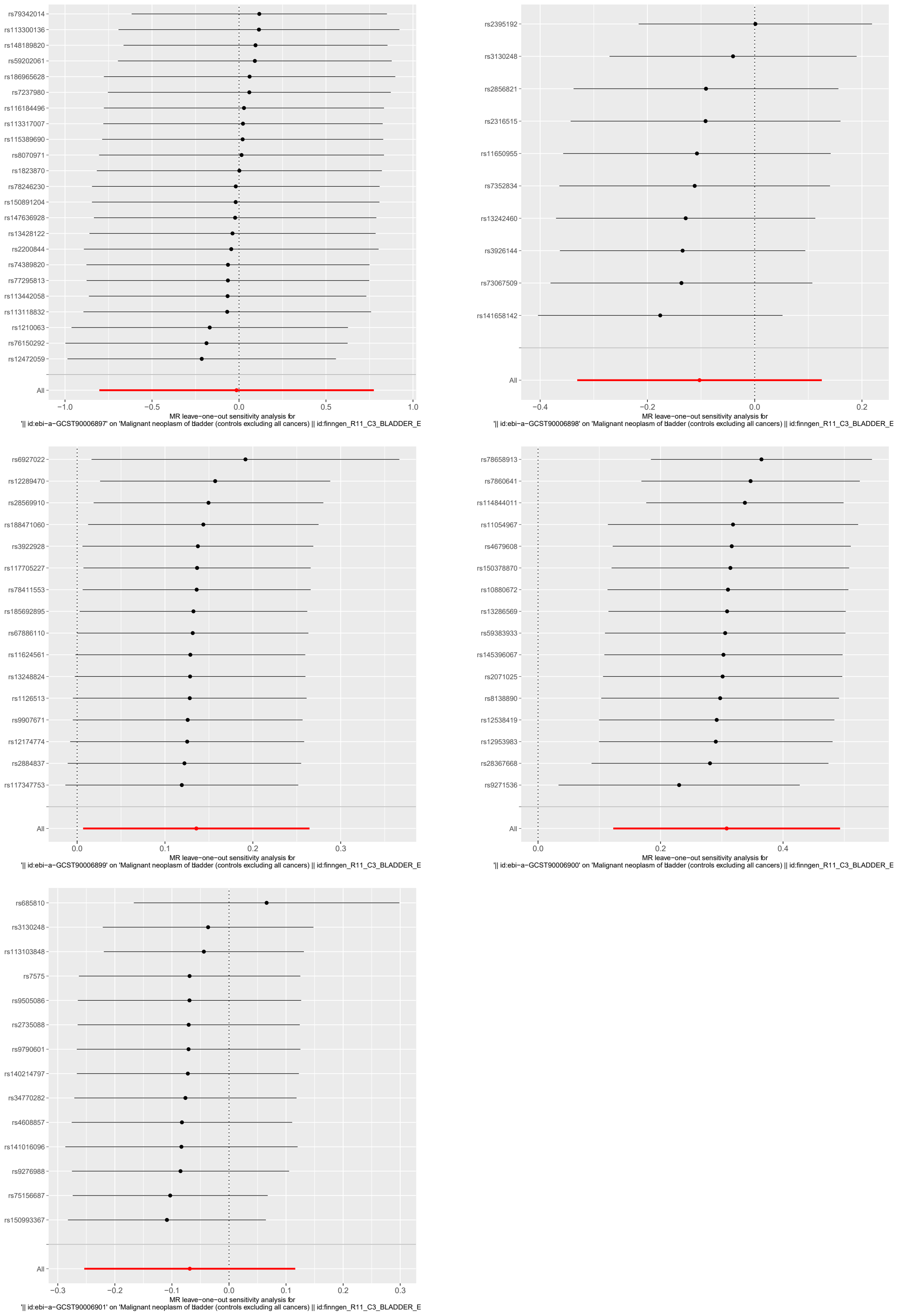

Scatterplot of BCa（R10）

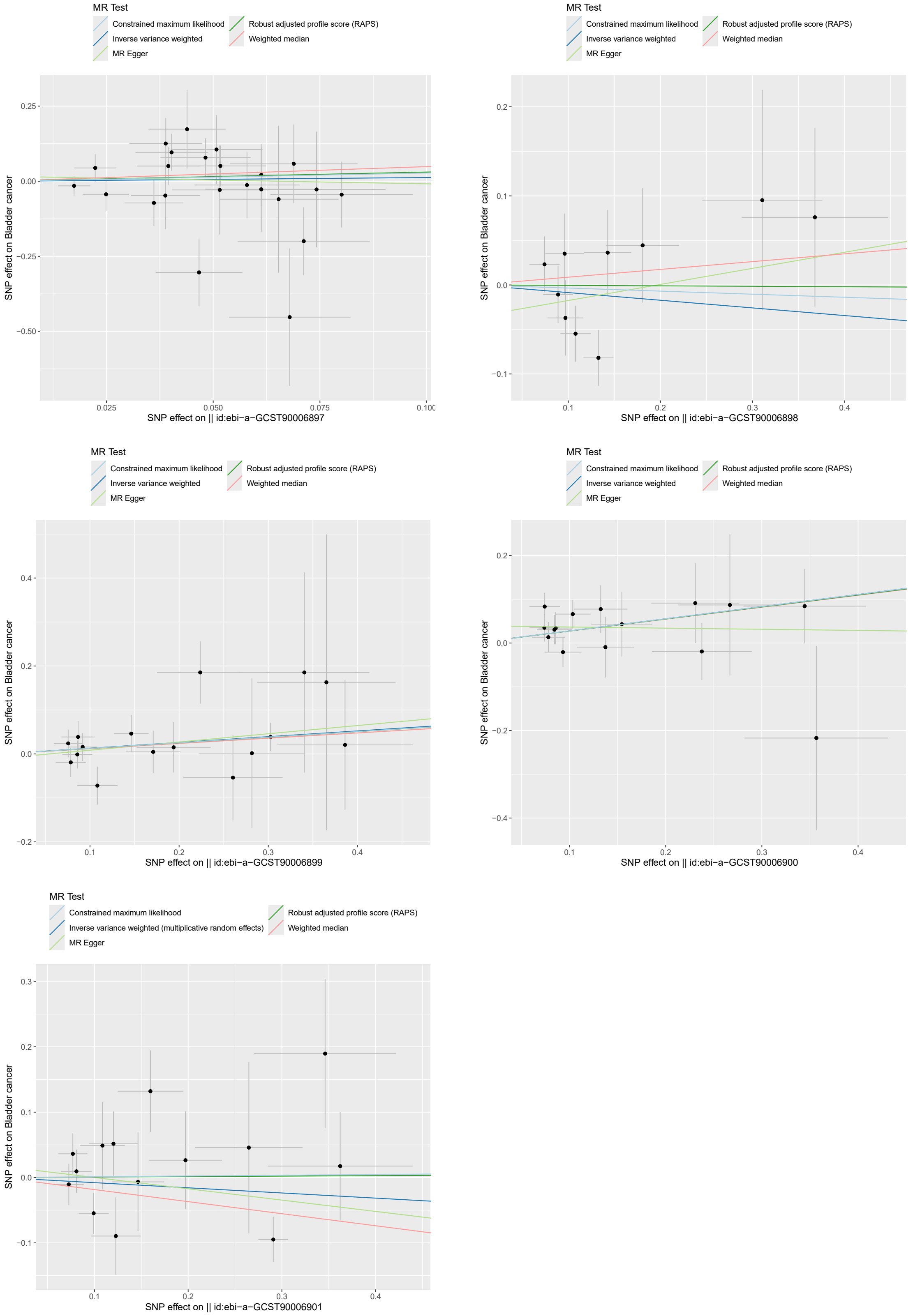

Forest map of BCa（R10）

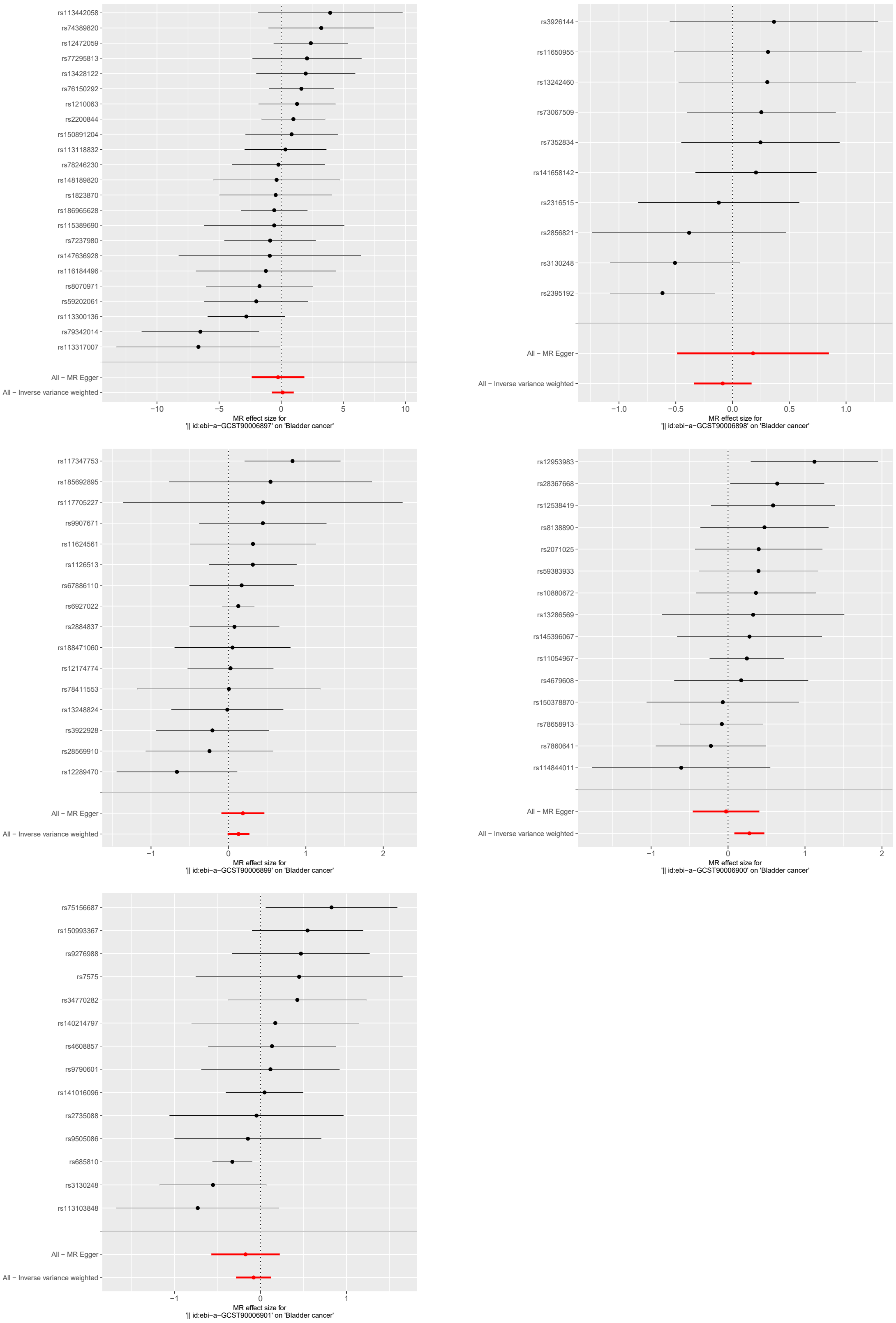

funnel plot of BCa（R10）

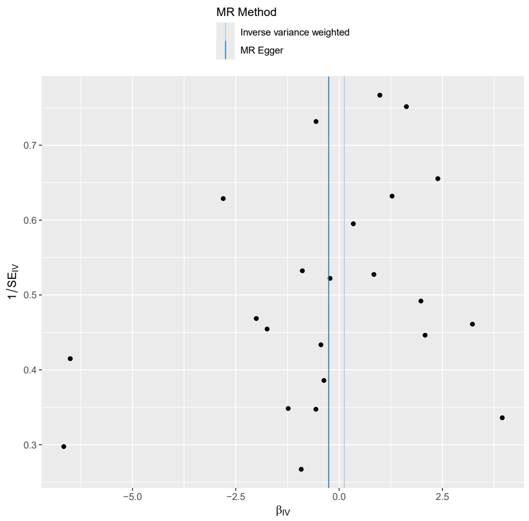

AEB-IgG

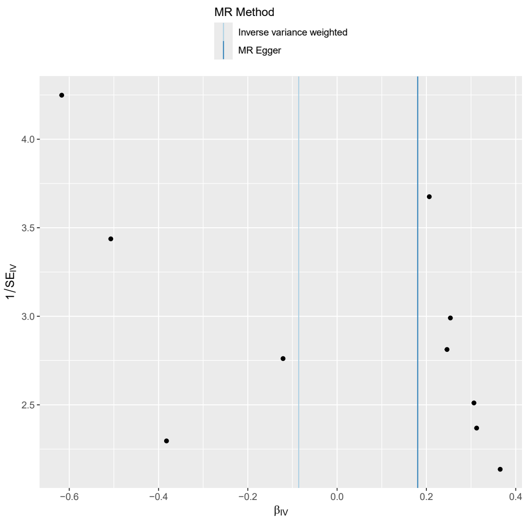

EA-D

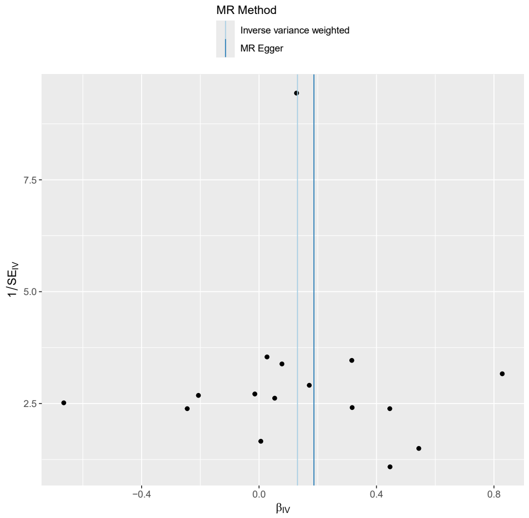

EBNA-1

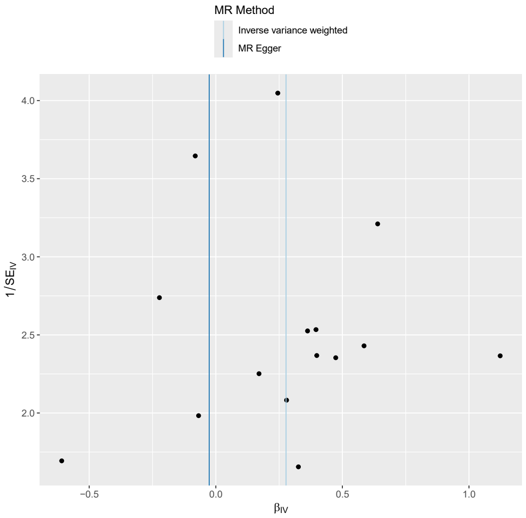

VCA-p18

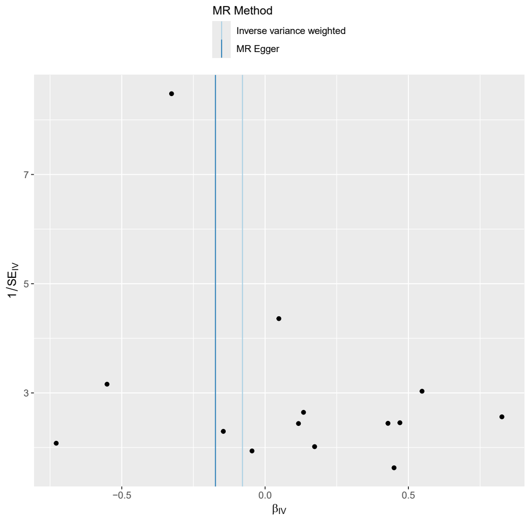

ZEBRA

leave-one-out of BCa（R10）

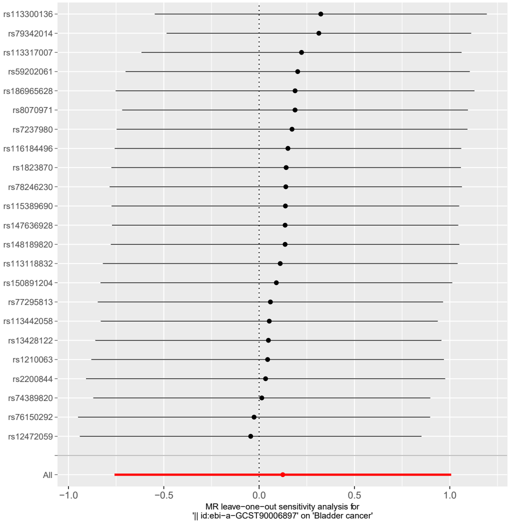

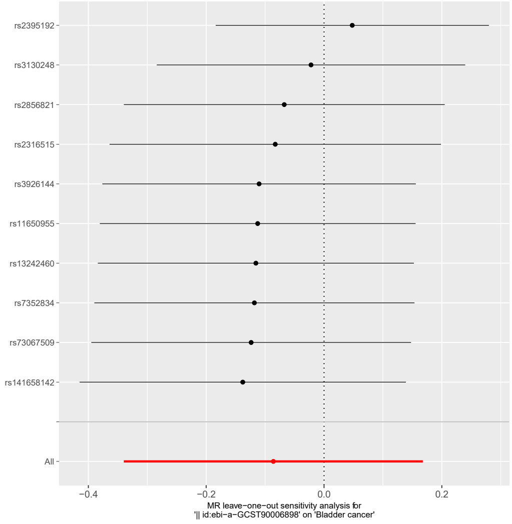

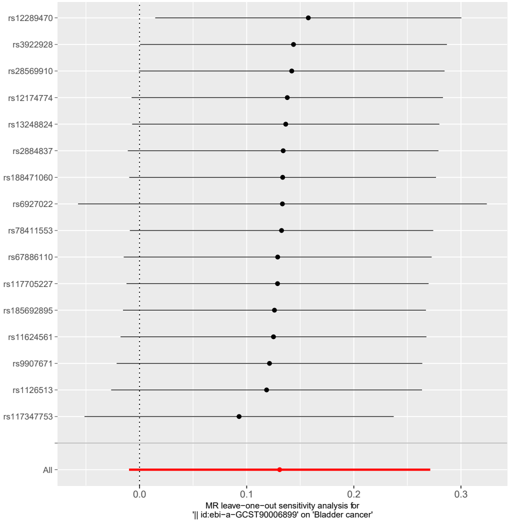

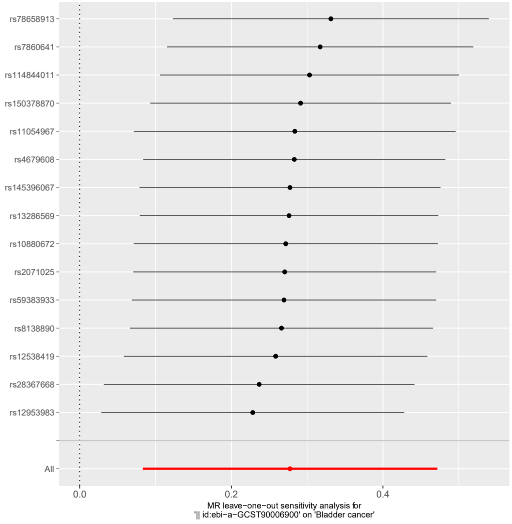

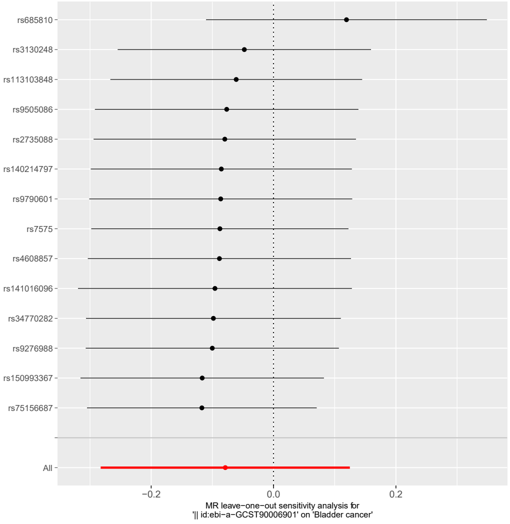

Reverse MR scatter plot

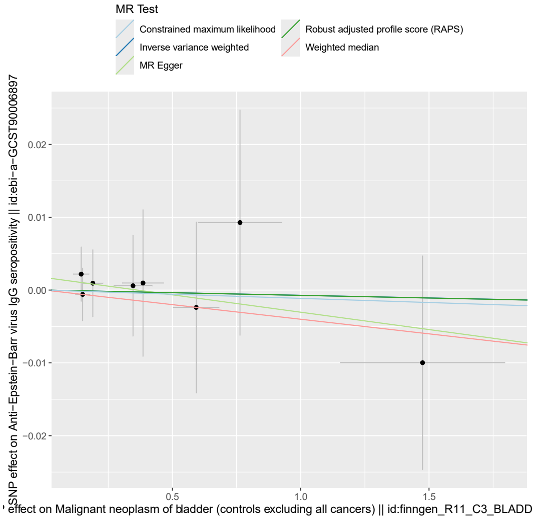

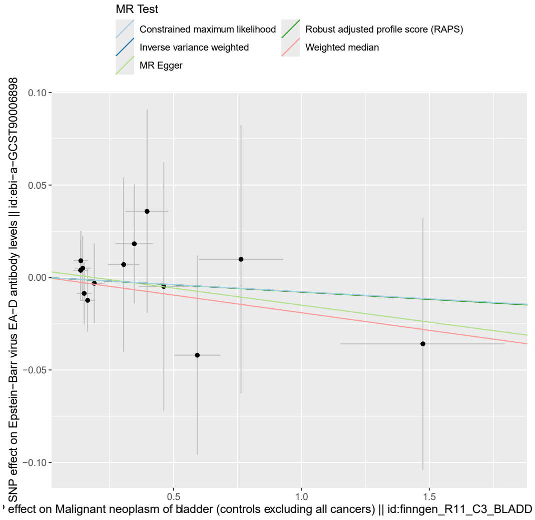

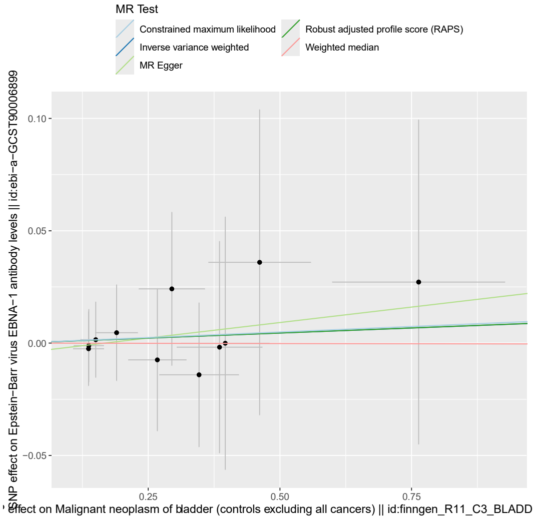

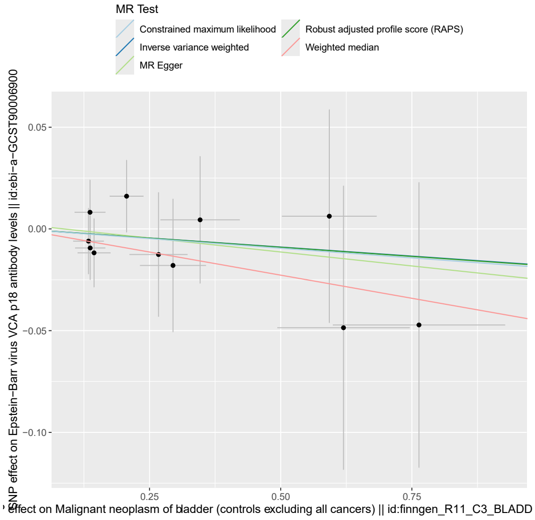

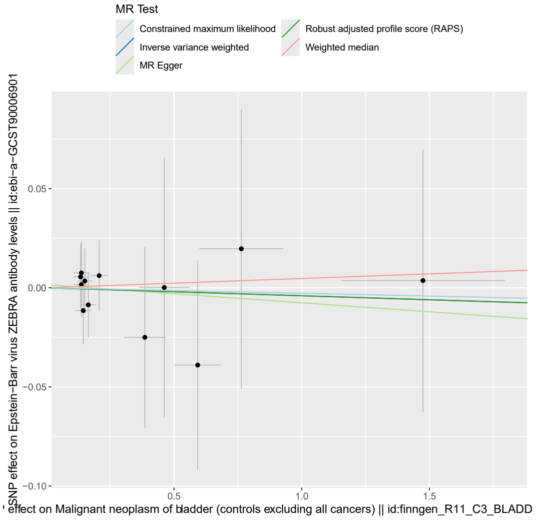

Reverse MR forest map

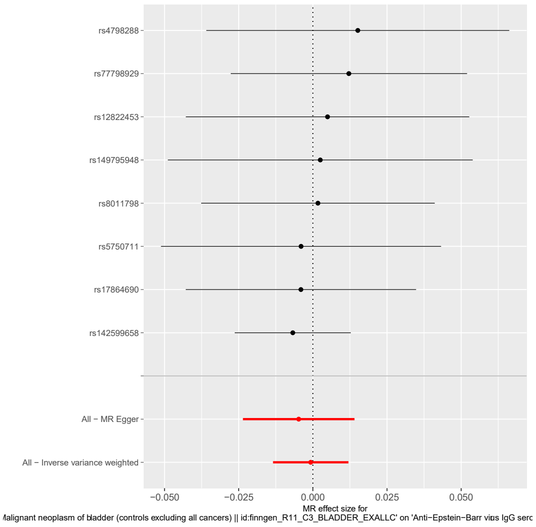

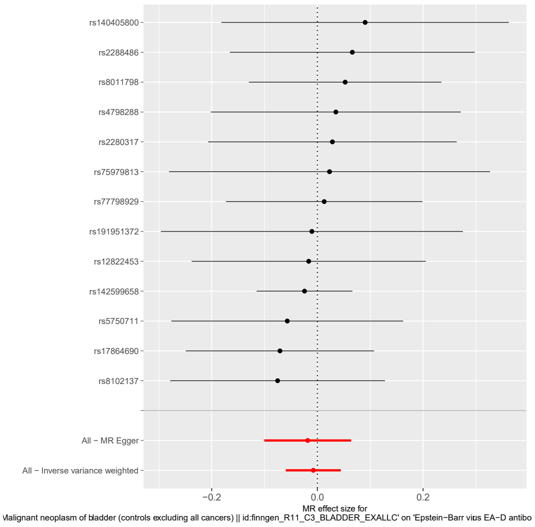

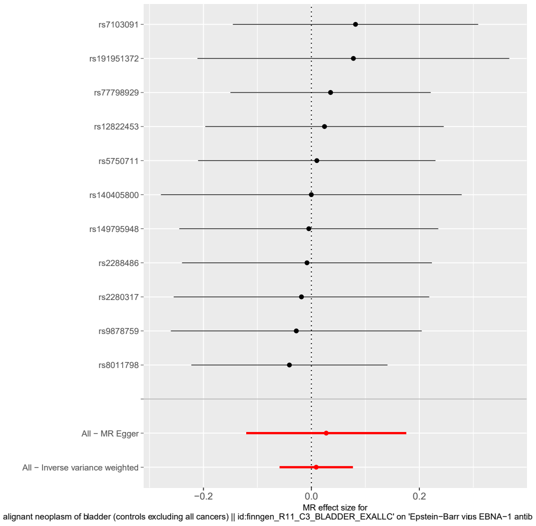

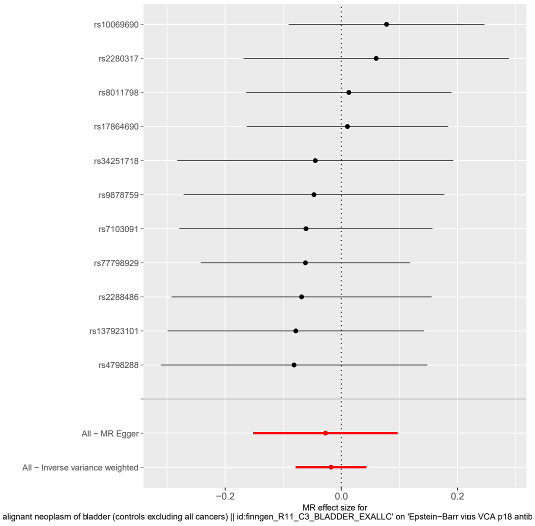

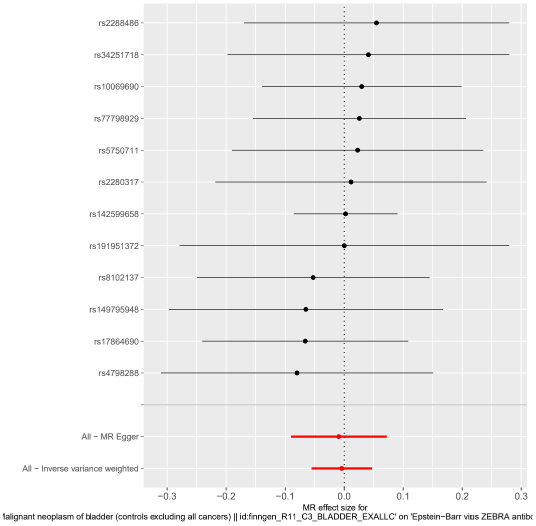

Reverse MR funnel plot

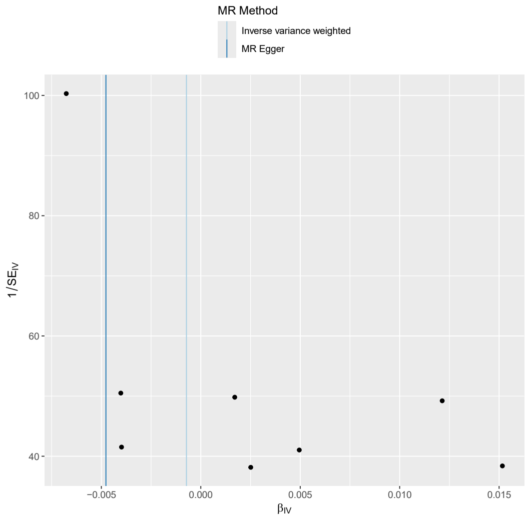

AEB-IgG

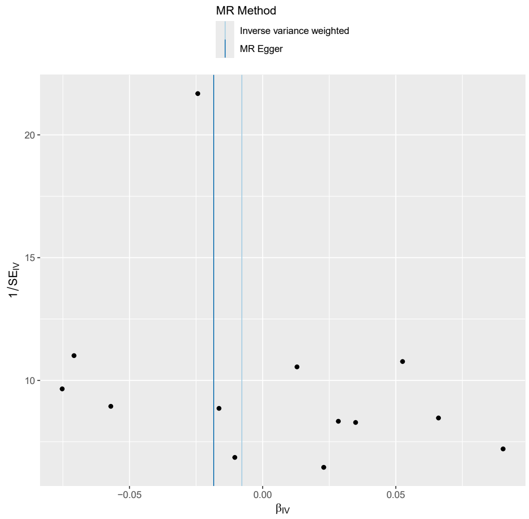

EA-D

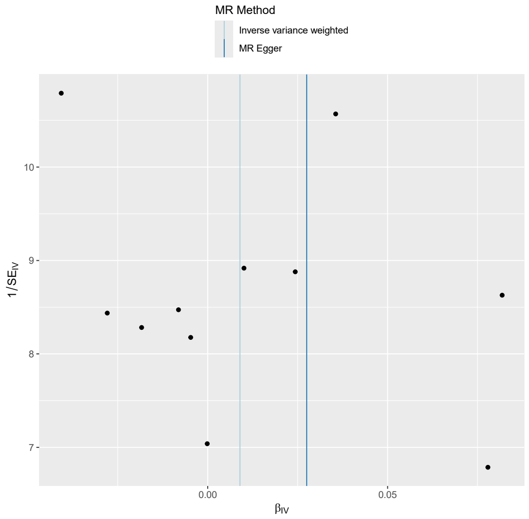

EBNA-1

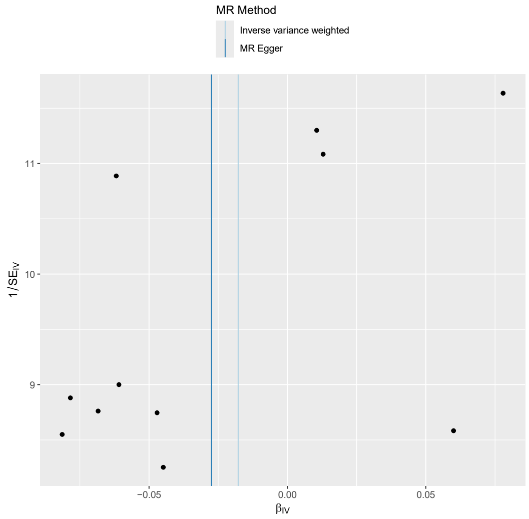

VCA-p18

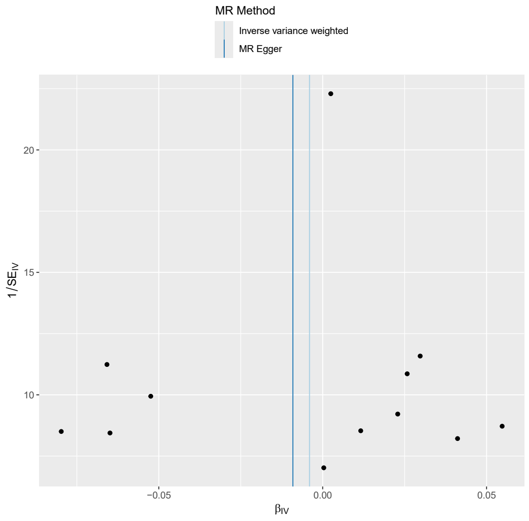

ZEBRA

leave-one-out of Reverse MR

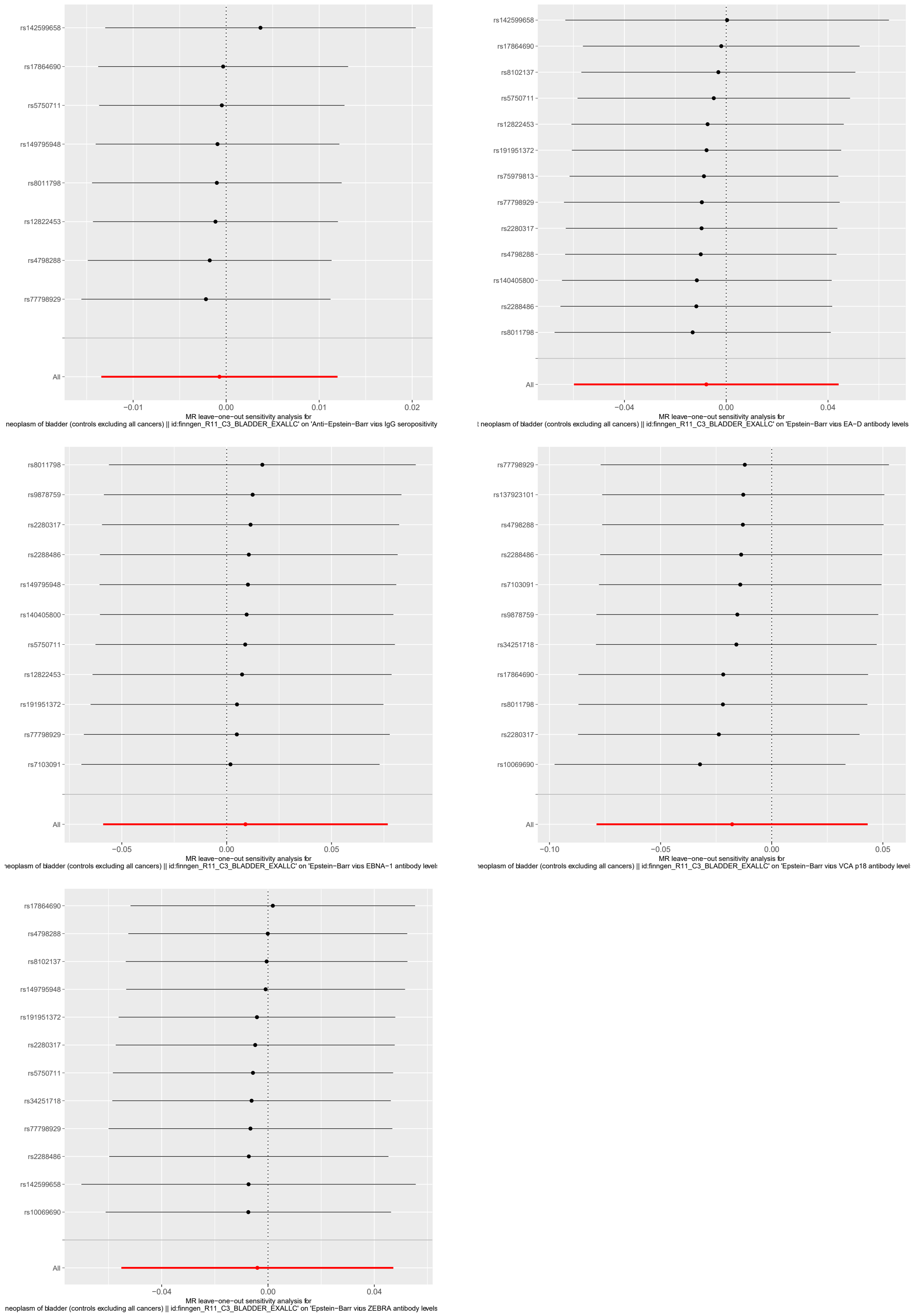

Supplement: Supplementary file 2 — Supplementary Material 2 [file 41598_2025_91594_MOESM2_ESM.pdf]
